# PKM2/STAT1-mediated PD-L1 upregulation on neutrophils during sepsis promotes neutrophil organ accumulation by serving an anti-apoptotic role

**DOI:** 10.1186/s12950-023-00341-2

**Published:** 2023-05-02

**Authors:** Yinjiaozhi Li, Ruoming Tan, Ranran Li, Rui Tian, Zhaojun Liu, Xiaoli Wang, Erzhen Chen, Tingting Pan, Hongping Qu

**Affiliations:** 1 Department of Critical Care Medicine, Ruijin Hospital, Shanghai Jiao Tong University School of Medicine, 197 Ruijin Er Road, Shanghai, 200025 China; 2 Department of Emergency Medicine, Ruijin Hospital, Shanghai Jiao Tong University School of Medicine, 197 Ruijin Er Road, Shanghai, 200025 China

**Keywords:** Sepsis, Neutrophil, Apoptosis, PD-L1, Glycolysis, PKM2

## Abstract

**Background:**

Delayed neutrophil apoptosis during sepsis may impact neutrophil organ accumulation and tissue immune homeostasis. Elucidating the mechanisms underlying neutrophil apoptosis may help identify potential therapeutic targets. Glycolysis is critical to neutrophil activities during sepsis. However, the precise mechanisms through which glycolysis regulates neutrophil physiology remain under-explored, especially those involving the non-metabolic functions of glycolytic enzymes. In the present study, the impact of programmed death ligand-1 (PD-L1) on neutrophil apoptosis was explored. The regulatory effect of the glycolytic enzyme, pyruvate kinase M2 (PKM2), whose role in septic neutrophils remains unaddressed, on neutrophil PD-L1 expression was also explored.

**Methods:**

Peripheral blood neutrophils were isolated from patients with sepsis and healthy controls. PD-L1 and PKM2 levels were determined by flow cytometry and Western blotting, respectively. Dimethyl sulfoxide (DMSO)-differentiated HL-60 cells were stimulated with lipopolysaccharide (LPS) as an in vitro simulation of septic neutrophils. Cell apoptosis was assessed by annexin V/propidium iodide (annexin V/PI) staining, as well as determination of protein levels of cleaved caspase-3 and myeloid cell leukemia-1 (Mcl-1) by Western blotting. An in vivo model of sepsis was constructed by intraperitoneal injection of LPS (5 mg/kg) for 16 h. Pulmonary and hepatic neutrophil infiltration was assessed by flow cytometry or immunohistochemistry.

**Results:**

PD-L1 level was elevated on neutrophils under septic conditions. Administration of neutralizing antibodies against PD-L1 partially reversed the inhibitory effect of LPS on neutrophil apoptosis. Neutrophil infiltration into the lung and liver was also reduced in PD-L1^−/−^ mice 16 h after sepsis induction. PKM2 was upregulated in septic neutrophils and promoted neutrophil PD-L1 expression both in vitro and in vivo. In addition, PKM2 nuclear translocation was increased after LPS stimulation, which promoted PD-L1 expression by directly interacting with and activating signal transducer and activator of transcription 1 (STAT1). Inhibition of PKM2 activity or STAT1 activation also led to increased neutrophil apoptosis.

**Conclusion:**

In this study, a PKM2/STAT1-mediated upregulation of PD-L1 on neutrophils and the anti-apoptotic effect of upregulated PD-L1 on neutrophils during sepsis were identified, which may result in increased pulmonary and hepatic neutrophil accumulation. These findings suggest that PKM2 and PD-L1 could serve as potential therapeutic targets.

**Supplementary Information:**

The online version contains supplementary material available at 10.1186/s12950-023-00341-2.

## Background

Sepsis, a syndrome caused by a dysregulated host response to infection, is characterized by both high in-hospital mortality and an unfavorable long-term prognosis [1]. Currently, there are no specific therapeutic measures for sepsis other than supportive strategies [2]. Immune dysregulation plays a key role in the pathogenesis of sepsis. Hyperinflammatory and immunosuppressive mechanisms often occur concurrently in patients with sepsis, involving phenotypic alterations in diverse immune cell types [3]. Neutrophils are the earliest-responding leukocyte population during infections [4], playing a crucial role in the antimicrobial response through rapid recruitment to infection sites. At the site of infection, these cells carry out a series of effector functions including phagocytosis, oxidative burst and the release of extracellular traps [5]. During sepsis, the circulating neutrophil population is significantly expanded, partly due to increased bone marrow mobilization [6]. And more importantly, a considerable number of studies demonstrated that neutrophil apoptosis is delayed during sepsis [7, 8]. Apoptosis is a critical pathway for the clearance of aged neutrophils, or neutrophils which have full-filled their bactericidal functions (e.g. phagocytosis) in the circulation and various tissues [9, 10]. Although an expanded neutrophil population may contribute to the improved elimination of pathogens, without proper clearance through apoptosis, activated neutrophils can over-accumulate in vital organs, causing tissue injury through the local release of antimicrobial substances and inflammatory mediators [11, 12]. Eventually, this over-accumulation caused by dysregulated apoptosis may result in the development of sepsis-induced multi-organ failure. Thus, gaining more mechanistic insight on neutrophil apoptosis during sepsis may provide new approaches to maintain tissue homeostasis during sepsis.

The interaction between the inhibitory checkpoint receptor, programmed death-1 (PD-1), and its ligands, programmed death ligands (PD-Ls), is recognized as a negative regulator of immune cell activation by attenuating signals from T cell receptors and CD28 [12, 13]. The biological effect of the PD-1/PD-L interaction has been extensively explored in the regulation of anti-tumor immune responses. This interaction is also implicated in the maintenance of immune tolerance versus autoimmunity, and T cell exhaustion during chronic inflammation [14–17]. Sepsis and cancer share the characteristic of immunosuppression, which makes it of comsiderable significance to improve our understanding of the immunoregulatory role of the PD-1/PD-L axis during sepsis. As a ligand for PD-1, PD-L1 is expressed on lymphocytes and antigen-presenting cells, as well as non-hematopoietic tissues like the cardiac endothelium, placenta, and pancreatic islets. Neutrophils also express PD-L1 [18]. Exploring the potential effect of PD-L1 on neutrophil activities and the regulatory mechanisms of neutrophil PD-L1 expression may provide further insights into the maintenance of immune homeostasis during sepsis.

In recent years, metabolic reprogramming has been proposed as a critical determinant of the functional fates of various innate and adaptive immune cells under infectious and inflammatory conditions [19]. Distinct from other leukocytes, neutrophils harbor few mitochondria. They rely on glycolysis as their major source of ATP and undergo gluconeogenesis to maintain intracellular glucose availability [20]. Glycolysis is fundamental to neutrophil bactericidal activities because neutrophil effector functions (e.g., phagocytosis and chemotaxis) have high ATP demands [21]. Inappropriately enhanced glycolysis can also cause abnormal neutrophil persistence and tissue injury [22]. In addition to providing the energy and biomolecules required for anabolic processes, the metabolic shift towards glycolysis also induces the upregulation of multiple enzymes and intermediaries with non-metabolic functions. The glycolytic metabolite, lactate, is shown to participate in signaling pathways and induce the post-transcriptional modifications of proteins [23, 24]. Glycolytic enzymes including hexokinase 2 (HK2), glyceraldehyde-3-phosphate dehydrogenase (GAPDH), α-enolase, lactate dehydrogenase A (LDHA), and pyruvate kinase M2 (PKM2) also regulate inflammatory responses in a metabolic-independent manner, interfering with the transcription process by binding to mRNA or interacting with transcription factors [25]. However, both the mechanism by which glycolysis modulates neutrophil essential functions and the potential regulatory effect of the non-metabolic functions of glycolytic enzymes on neutrophil activities remain under-explored in the context of sepsis.

This study provides evidence of the essential role of PKM2, a glycolytic enzyme that has attracted recent attention due to its non-metabolic functions, on neutrophil PD-L1 upregulation during sepsis, which in turn enhances neutrophil accumulation in vital organs by inhibiting neutrophil apoptosis. Mechanistically, PKM2 translocates to the nucleus and leads to signal transducer and activator of transcription 1 (STAT1) activation, which promotes transcription of the STAT1 target gene, PD-L1. The findings highlight the regulatory mechanism of PD-L1 expression on neutrophils during sepsis. This involves the non-metabolic role of the glycolytic enzyme, PKM2, which provides potential targets to modulate tissue inflammation by controlling neutrophil accumulation in vital organs during sepsis.

## Results

### Peripheral blood neutrophils from patients with sepsis exhibited upregulated surface levels of PD-L1

**Fig. 1:**
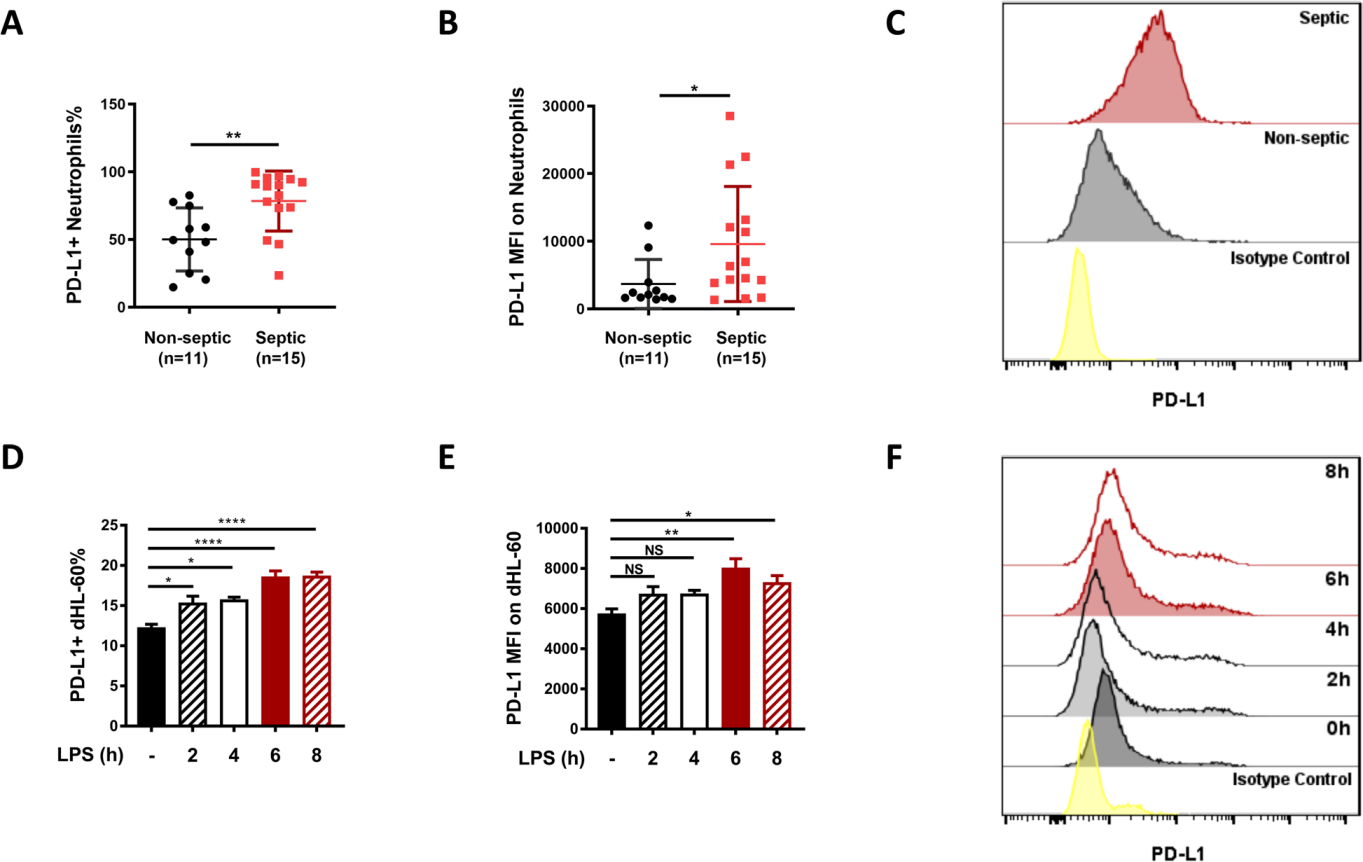
Peripheral neutrophils exhibit upregulated surface level of PD-L1 during sepsis. **A**, **B** Peripheral blood neutrophils were isolated from septic patients and healthy controls. Proportion of PD-L1^+^ neutrophils (**A**) and MFI of PD-L1 on neutrophils (**B**) were determined by flow cytometry. **C** Representative flow cytometry image of PD-L1 levels on neutrophils from septic patients and healthy controls. **D**, **E** An in vitro simulation of neutrophils under septic condition was established by LPS (1ug/ml) stimulation of dHL-60 cells for 2 h, 4 h, 6 h and 8 h (*n* = 4). Proportion of PD-L1^+^ dHL-60s (**D**) and MFI of PD-L1 on dHL-60s (**E**) were determined by flow cytometry. **F** Representative flow cytometry image of PD-L1 levels on dHL-60s subjected to different durations of LPS stimulation. Mann Whitney test and one-way ANOVA were performed. Data are presented as the mean ± SEM. *P < 0.05; **P < 0.01; ****P < 0.0001. NS, not significant

To explore the implication of PD-L1 expression in neutrophil physiology during sepsis, surface levels of neutrophil PD-L1 were assessed in both patients with sepsis and healthy controls. Peripheral neutrophils were isolated from 15 septic patients and 11 healthy volunteers and PD-L1 levels were quantified by flow cytometry. The general characteristics of patients enrolled are shown in Table 1. Surface levels of PD-L1 were significantly elevated in neutrophils from patients with sepsis compared to those from healthy volunteers as measured by both the proportion of PD-L1^+^ neutrophils (78.4 ± 22.2% vs. 50.0 ± 23.3%, respectively; *P* < 0.01) and the mean fluorescence intensity (MFI) of PD-L1 on neutrophils (6333, IQR: 3834–13,194 vs. 2116, ± IQR: 1641–3927; *P* < 0.05) (Fig. 1A-C). For subsequent experiments, an in vitro model of neutrophils under septic conditions was established through lipopolysaccharide (LPS) stimulation of neutrophil-like, dimethyl sulfoxide (DMSO)-differentiated HL-60 cells (dHL-60s). The upregulation of PD-L1 was recaptured in this model as shown by a significantly higher positive rate and MFI of PD-L1 on cells stimulated with LPS for 6 h compared to unstimulated cells (Fig. 1D-F). Gating strategies for peripheral neutrophils and dHL-60 s in the above two experiments are shown in Supplementary Fig. 2.

**Table 1:** General characteristics of septic patients enrolled

Items	Value^a^
Sex (male/female)	9/6
Age (years)	66 (61–77)
Infection site	
Peritoneal cavity	4
Respiratory system	4
Blood stream infection	1
Multisite infection	6
Severity Score	
APACHE II^b^	19 (13–23)
SOFA^c^	6 (4–9)
Length of stay in ICU^d^ (days)	11 (5–41)
Outcome (survived/dead)	14/1

^a^ Continuous data presented as median (inter-quartile range)

^b^ *APACHE II* Acute physiology and chronic health evaluation II

^c^ *SOFA* Sequential organ failure assessment

^d^ *ICU* Intensive care unit

### Upregulated PD-L1 inhibited neutrophil apoptosis, leading to increased pulmonary and hepatic neutrophil accumulation in a sepsis animal model

**Fig. 2:**
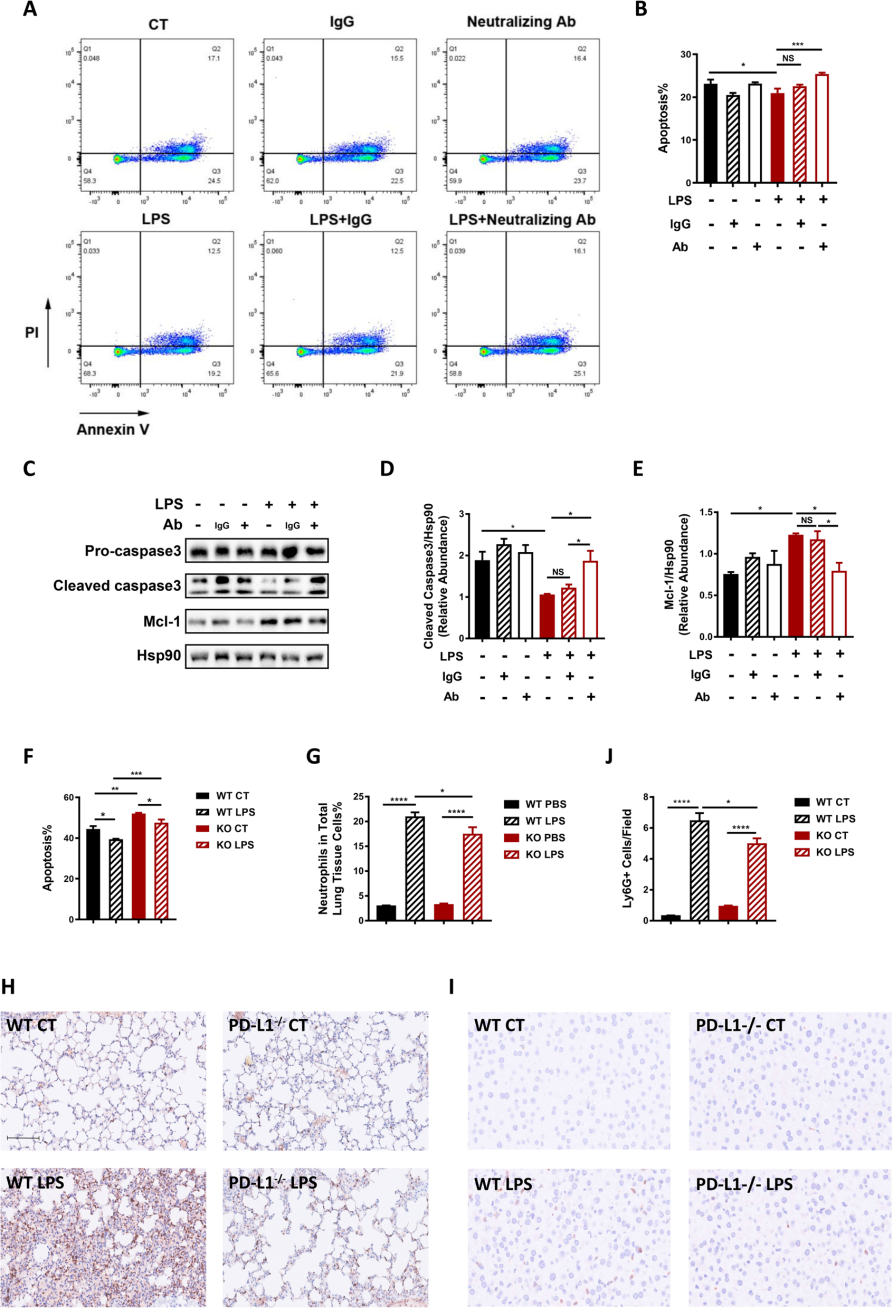
PD-L1 inhibits neutrophil apoptosis and increases sepsis-induced neutrophil accumulation in the lung and liver. **A**-**E** dHL-60 s were treated with LPS (1ug/ml), pretreated with a PD-L1 neutralizing antibody (InVivoMAb anti-human PD-L1 (B7-H1), Bioxcell, Lebanon, NH, USA) (20ug/ml) 0.5 h before LPS stimulation, or pretreated with an isotype control for the neutralizing antibody (InVivoMAb mouse IgG2b isotype control, Bioxcell, Lebanon, NH, USA) (20ug/ml) 0.5 h before LPS stimulation. **A**, **B** dHL-60s from different treatment groups were stained with annexin V/PI 24 h after LPS stimulation (*n* = 3). **A** Representative flow cytometry images for apoptotic dHL-60s. **B** Histogram for the proportions of apoptotic cells in different treatment groups. **C**-**E** Levels of apoptosis-related proteins of dHL-60s from different treatment groups were analyzed by Western blotting. Hsp90 was used as a loading control. The blot is representative of three independent experiments. **F** Bone marrow neutrophils isolated from wild type and PD-L1^−/−^ C57BL/6 J mice were stimulated with LPS ex vivo for 24 h. Proportions of apoptotic cells in each group were determined by flowcytometry (*n* = 4). **G**-**J** Pulmonary and hepatic neutrophil infiltration were assessed in wild type and PD-L1^−/−^ C57BL/6 J mice subjected to sepsis induction. **G** Histogram of the proportion of CD45^+^CD11b^+^Ly6G^+^ cells in the total cells of the right lung lobe (*n* = 8 each group). **H** Representative immunohistochemistry images of Ly6G^+^ cells in the lung section. Scale bar: 100uM. **I** Representative immunohistochemistry images of Ly6G + cells in the liver section. Scale bar: 100uM. **J** Histogram of the number of Ly6G^+^ cells per 400 × field of the liver sections (*n* = 6 each group; 10 random selected felds were analyzed per section). One-way ANOVA was performed. Data are presented as the mean ± SEM. *P < 0.05; **P < 0.01; ***P < 0.001; ****P < 0.0001. NS, not significant

In contrast to the well-established role of neutrophil PD-L1 in T cell exhaustion by binding with PD-1 on T cells [26, 27], the intrinsic impact of PD-L1 expression on PD-L1^+^ neutrophils hasn’t been widely explored. Apoptosis is critical for clearing neutrophils and resolving neutrophil-mediated inflammation [28]. Thus, the effect of PD-L1 on dHL-60 apoptosis under LPS stimulation was examined by blocking PD-L1 with a neutralizing antibody. Reduced apoptosis of dHL-60s under LPS stimulation was observed compared to untreated dHL-60s, as evidenced by reduced proportions of annexin V^+^ cells (Fig. 2A, B), decreased levels of cleaved caspase-3, and increased levels of the anti-apoptotic protein, Mcl-1 (Fig. 2C-E). Pretreatment with PD-L1 neutralizing antibody before LPS stimulation significantly increased dHL-60 apoptosis compared to pretreatment with the isotype-control version of the neutralizing antibody (Fig. 2A-E), indicating an anti-apoptotic role of PD-L1 on LPS-stimulated dHL-60 s. Isolated bone marrow neutrophils from PD-L1-/- mice also exhibited higher apoptotic rate after LPS stimulation compared to wild type mice (Fig. 2F). To determine the in vivo implication of this anti-apoptotic effect of neutrophil PD-L1, sepsis was induced in wild type and PD-L1^−/−^ C57BL/6 J mice by intraperitoneal LPS injection. Induction of sepsis increased the proportion of CD45^+^CD11b^+^Ly6G^+^ cells in the lung tissue (Fig. 2G) and increased the density of Ly6G^+^ cells in lung sections, compared to phosphate buffered saline (PBS)-treated control mice (Fig. 2H). This was observed in both wild type and PD-L1^−/−^ mice. However, PD-L1^−/−^ mice showed significantly reduced pulmonary neutrophil accumulation than wild type mice (Fig. 2G, H) 16h after sepsis induction. A consistent trend was observed in the liver, as immunohistochemical staining of liver sections also revealed fewer Ly6G^+^ cells in the liver of PD-L1^−/−^ mice compared to those of wild type mice 16h after sepsis induction (Fig. 2I, J). Together, these results suggest that PD-L1 expression prolongs neutrophil survival and may be responsible for neutrophil accumulation in vital organs during sepsis.

### PKM2 was responsible for neutrophil PD-L1 upregulation and reduced apoptosis during sepsis

**Fig. 3:**
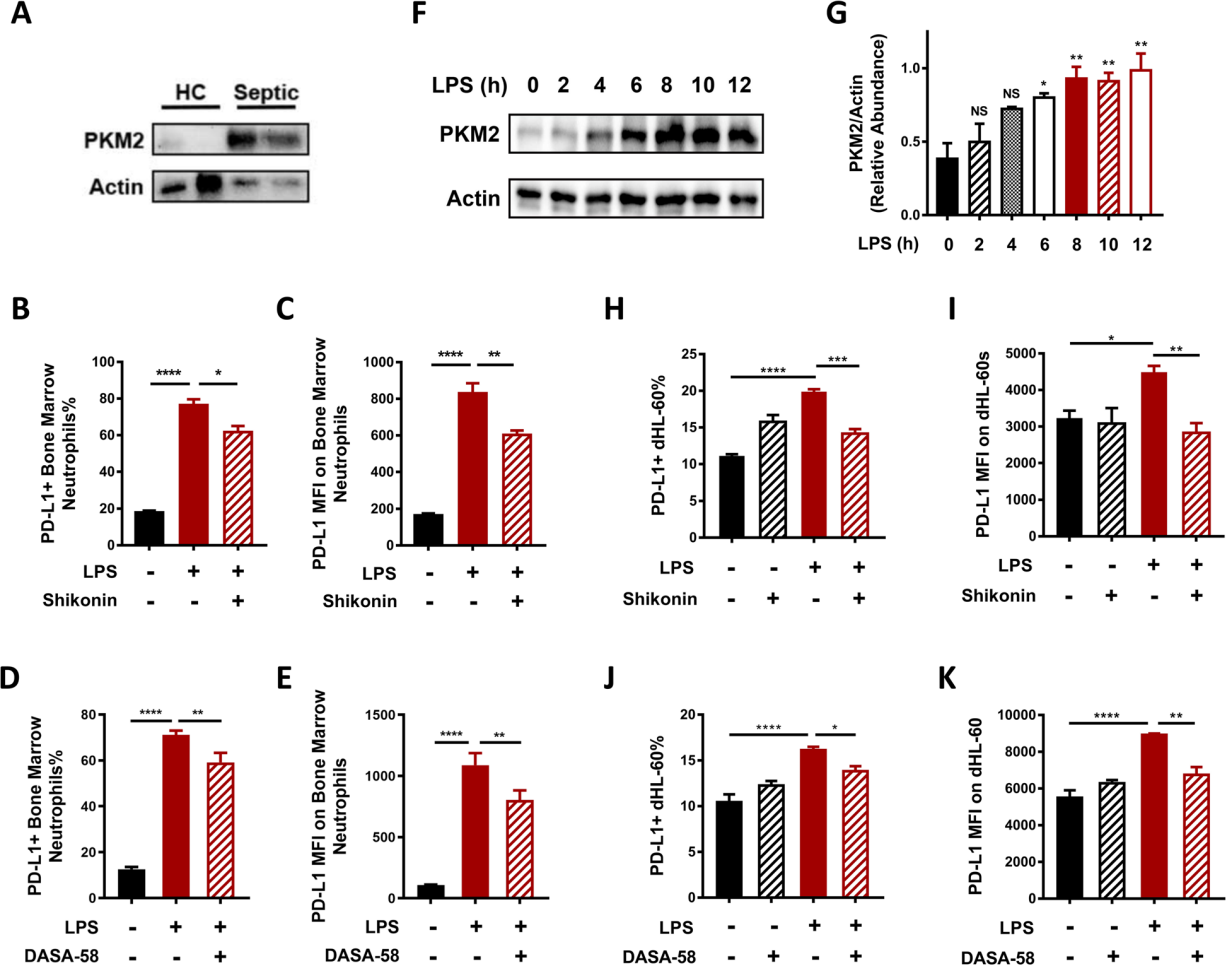
PKM2 activity, especially its nuclear translocation, promotes neutrophil PD-L1 expression during sepsis. **A** Protein levels of PKM2 in peripheral neutrophils from septic patients and healthy controls were analyzed by Western blotting. β-actin was used as the loading control. The blot is representative of three independent experiments. **B**, **C** C57BL/6 J mice were injected with PBS, LPS, or pretreated with shikonin (10 mg/kg) before LPS injection. PD-L1 levels on bone marrow neutrophils from mice under different treatments were determined by flow cytometry (**B**) and MFI of PD-L1 on bone marrow neutrophils (**C**) were measured (*n* = 3 each group). **D**, **E** C57BL/6 J mice were injected with PBS, LPS, or pretreated with DASA-58 (25 mg/kg) before LPS injection. PD-L1 levels on bone marrow neutrophils from mice under different treatments were determined by flow cytometry. Proportion of PD-L1^+^ bone marrow neutrophils (**D**) and MFI of PD-L1 on bone marrow neutrophils (**E**) were measured (*n* = 6 each group). **F**, **G** Protein levels of PKM2 in dHL-60s subjected to LPS treatment for 2 h, 4 h, 6 h, 8 h, 10 h and 12 h were determined by Western blotting. β-actin was used as the loading control. The blot is representative of three independent experiments. **H**, **I** PD-L1 levels of untreated dHL-60s and dHL-60s subjected to LPS treatment for 6 h, pretreatment with shikonin (1uM) before LPS stimulation, or shikonin treatment alone were measured by flow cytometry (*n* = 3). **J**, **K** PD-L1 levels of untreated dHL-60s and dHL-60s subjected to LPS treatment for 6 h, pretreatment with DASA-58 (20uM/ml) before LPS stimulation, or DASA-58 treatment alone were measured by flow cytometry (*n* = 4). One-way ANOVA was performed. Data are presented as the mean ± SEM. *P < 0.05; **P < 0.01; ***P < 0.001; ****P < 0.0001. NS, not significant

A metabolic shift toward glycolysis involves the altered expression of genes encoding glycolytic enzymes [29]. As one of those enzymes, PKM2 has drawn much attention since it possesses not only catalytic activity but also non-metabolic functions, which have been implicated in immune regulation during infectious and autoimmune conditions [30]. Peripheral neutrophils from patients with sepsis showed significantly higher protein levels of PKM2 compared to those from healthy volunteers (Fig. 3A), which led us to speculate a role for PKM2 in the regulation of PD-L1 expression in septic neutrophils. This hypothesis was first tested in an in vivo setting by pretreating mice with shikonin, a potent inhibitor of PKM2 derived from *Lithospermum erythrorhizon* [30], prior to sepsis induction. PD-L1 levels were significantly increased on bone marrow neutrophils 16 h after LPS treatment, while shikonin pretreatment significantly attenuated PD-L1 upregulation induced by LPS (Fig. 3B, C). Pretreatment with another compound, DASA-58, which inhibits nuclear translocation of PKM2 by promoting the formation of PKM2 tetramers [31], before sepsis induction, yielded the same effect as pretreatment with shikonin. In dHL-60s subjected to LPS stimulation, the protein levels of PKM2 also showed a time-dependent increase (Fig. 3F, G), which is compatible with the increase in PD-L1 levels. We also pretreated dHL-60s with shikonin and DASA-58 before LPS stimulation. As a result, both compounds reduced the level of PD-L1 under LPS stimulation as measured by both the proportion of PD-L1^+^ dHL-60s and MFI of PD-L1 on dHL-60s (Fig. 3H-K). These results indicate that PKM2 activity, especially its nuclear translocation, is required for PD-L1 upregulation in LPS stimulated dHL-60s.

**Fig. 4:**
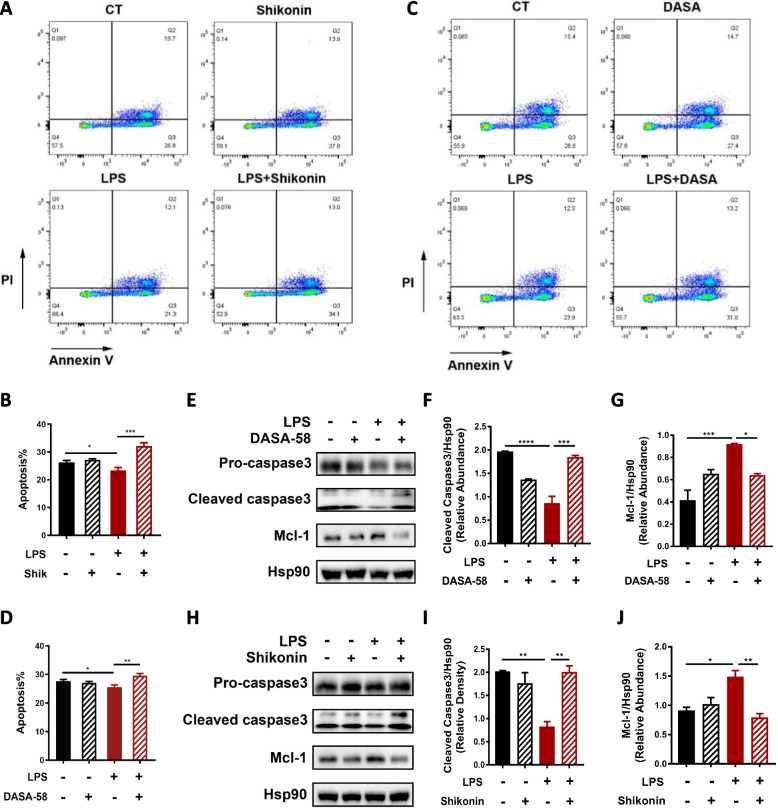
PKM2 expression and nuclear translocation inhibit dHL-60 apoptosis under LPS stimulation. dHL-60s were subjected to LPS treatment, pretreatment with shikonin/DASA-58 before LPS stimulation, or shikonin/DASA-58 treatment alone. **A**-**D** dHL-60s were stained by annexin V/PI and assessed by flow cytometry. **A**, **C** Representative flow cytometry images of dHL-60 apoptosis. **B**, **D** Histograms of the apoptosis rates of dHL-60s subjected to different treatments (*n* = 3–4). **E**-**J** Levels of apoptosis-related proteins were determined by Western blotting. Hsp90 was used as the loading control. The blot is representative of three independent experiments. One-way ANOVA was performed. Data are presented as the mean ± SEM. *P < 0.05; **P < 0.01; ***P < 0.001; ****P < 0.0001

Given the anti-apoptotic role of PD-L1, the effect of PKM2 on dHL-60 apoptosis was examined. The proportion of apoptotic cells measured by flow cytometry (Fig. 4A-D), and protein levels of cleaved caspase-3 and Mcl-1 (Fig. 4E-J) both indicated increased apoptosis of dHL-60s with shikonin/DASA-58 pretreatment before LPS stimulation compared with those stimulated with LPS alone. These observations suggest that PKM2 inhibits neutrophil apoptosis potentially through promoting PD-L1 expression.

### PKM2 promoted PD-L1 upregulation through activation of the transcription factor STAT1

**Fig. 5:**
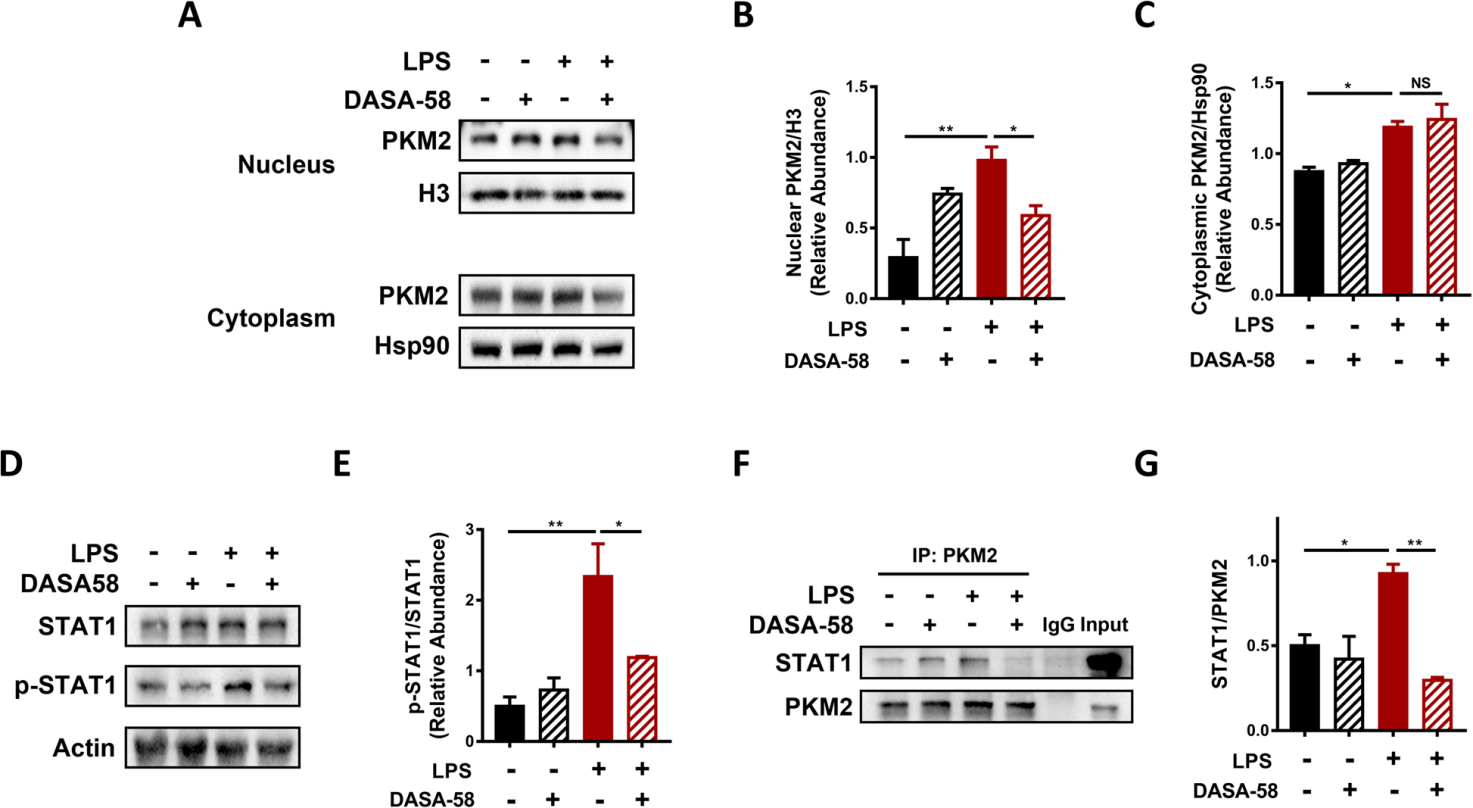
PKM2 translocates to the nucleus and activates STAT1 through direct interaction. dHL-60s were treated with LPS (1ug/ml) alone for 6 h, pretreated with DASA-58 (20uM) 0.5 h before LPS treatment or treated with DASA-58 alone. **A**-**C** Protein levels of PKM2 in the cytoplasm and nucleus of dHL-60s were determined by Western blotting. Hsp90 was used as the loading control for the cytoplasmic protein. Histone H3 was used as the loading control for the nuclear protein. The blot is representative of three independent experiments. **D**, **E** Protein level of p-STAT1 was determined by Western blotting. β-actin was used as the loading control. The blot is representative of three independent experiments. **F**, **G** Protein–protein interaction between PKM2 and STAT1 was determined by co-immunoprecipitation assay. The blot is representative of three independent experiments. One-way ANOVA was performed. Data are presented as the mean ± SEM. *P < 0.05; **P < 0.01

To gain mechanistic insights into the regulatory effect of PKM2 on PD-L1 expression, we first determined the intracellular localization of PKM2 in dHL-60s under LPS and DASA-58 treatment. LPS treatment induced a significant increase in both the cytoplasmic and nuclear levels of PKM2. DASA-58 pretreatment before LPS stimulation reversed the increase in nuclear levels of PKM2 induced by LPS but had no effect on the cytoplasmic level of PKM2 (Fig. 5A-C). PKM2 can interact with multiple transcription factors and affect the transcription of their target genes after nuclear translocation [32–34]. Among those transcription factors, the STAT family members, STAT1, STAT3, and STAT5, are known to be highly-specific with no need for secondary messengers once they are phosphorylated [35]. Furthermore, STAT1 phosphorylation was found to be indispensable for IFN-γ- and IL-27-induced PD-L1 expression on tumor cells and monocytes [36, 37]. Therefore, the impact of PKM2 nuclear translocation on STAT1 activation and the involvement of STAT1 activation in PKM2-mediated PD-L1 upregulation on septic neutrophils were examined. Phosphorylation of STAT1 at the Y701 site was significantly increased upon LPS stimulation of dHL-60s and this increase was mitigated by DASA-58 pretreatment (Fig. 5D, E), suggesting PKM2 nuclear translocation was involved in the increased phosphorylation of STAT1. In addition, the co-IP assay revealed that the PKM2-STAT1 protein–protein interaction was increased upon LPS stimulation and decreased by DASA-58 pretreatment (Fig. 5F, G), indicating phosphorylation of STAT1 by intra-nuclear PKM2 through direct interaction.

**Fig. 6:**
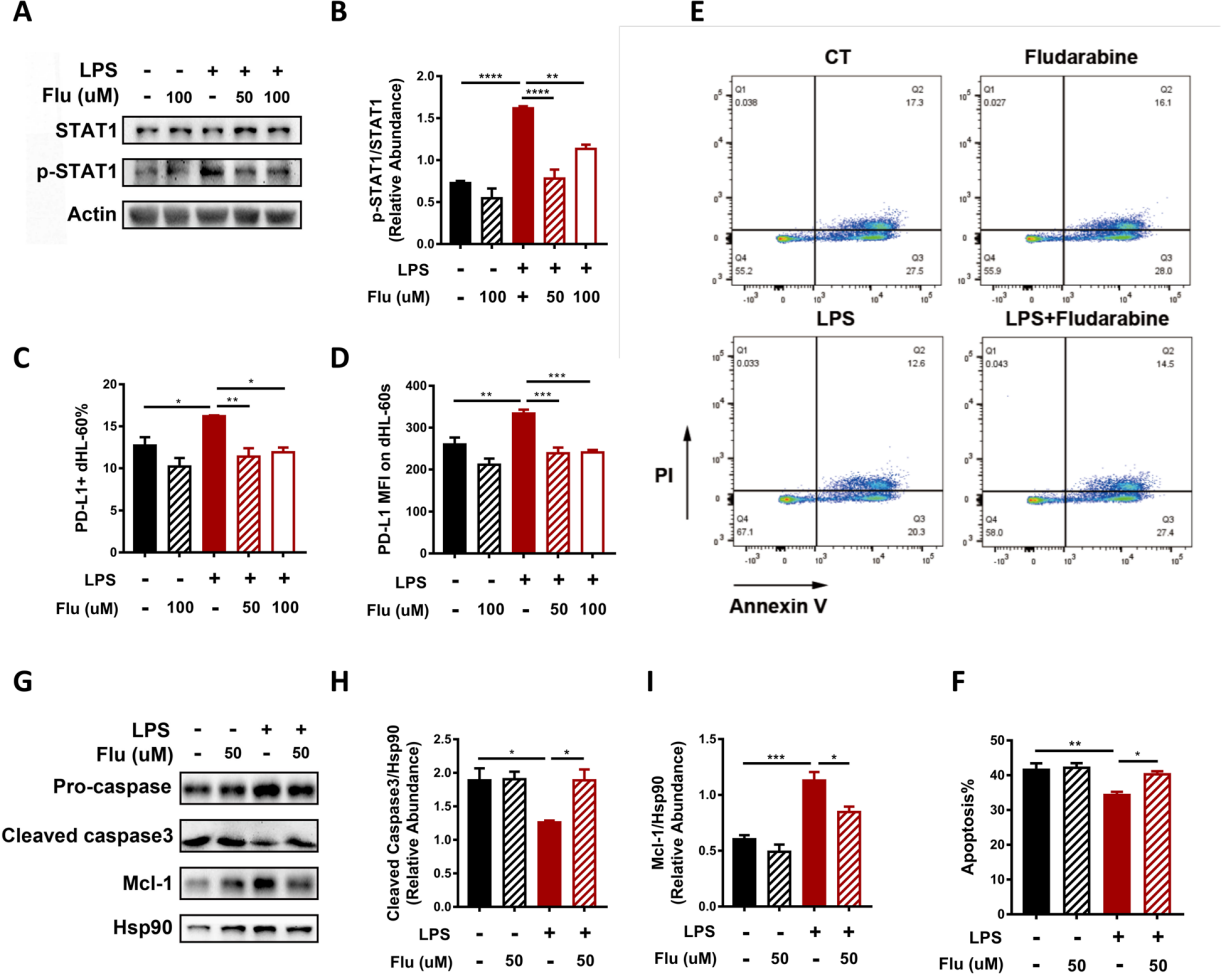
STAT1 activation promotes PD-L1 upregulation and inhibits dHL-60 apoptosis under LPS stimulation. **A**-**D** dHL-60s were treated with LPS (1ug/ml) alone for 6 h, pretreated with Fludarabine (50 and 100 uM) before LPS stimulation or treated with Fludarabine (100uM) alone. **A**, **B** Protein level of p-STAT1 was determined by Western blotting. β-actin was used as the loading control. The blot is representative of three independent experiments. **C**, **D** PD-L1 levels on dHL-60s under different treatments were measured by flow cytometry. Proportion of PD-L1^+^ dHL-60 s (**C**) and MFI of PD-L1 on dHL-60s (**D**) were measured (*n* = 3). **E**-**I** dHL-60 s were treated with LPS (1ug/ml) alone, pretreated with Fludarabine (50uM) before LPS stimulation or treated with Fludarabine (50uM) alone. **E**, **F** dHL-60s were stained by annexin V/PI and assessed by flowcytometry (*n* = 3). **E** Representative flow cytometry images of dHL-60 apoptosis. **F** Histogram of the apoptosis rates of dHL-60 s subjected to different treatments. **G**-**I** Levels of apoptosis-related proteins were determined by Western blotting. Hsp90 was used as the loading control. The blot is representative of three independent experiments. One-way ANOVA was performed. Data are presented as the mean ± SEM. *P < 0.05; **P < 0.01; ***P < 0.001; ****P < 0.0001

To gain direct support for the involvement of STAT1 in the PKM2-mediated upregulation of PD-L1, dHL-60s were pretreated with the specific inhibitor of STAT1 phosphorylation, fludarabine, before LPS stimulation [38, 39]. As shown in Fig. 6A and B, fludarabine pretreatment successfully prevented LPS-induced STAT1 phosphorylation. Compared to LPS treatment alone, pretreatment with fludarabine diminished the upregulation of PD-L1 as evidenced by a significant decrease in both the proportion of PD-L1^+^ dHL-60 s and the MFI of PD-L1 on dHL-60 s (Fig. 6C-D), suggesting that STAT1 activation was required for PD-L1 upregulation on neutrophils. In accordance with the anti-apoptotic effect of PD-L1, fludarabine pretreatment before LPS stimulation restored dHL-60 apoptosis, as evidenced by the results of flow cytometry (Fig. 6E-F) and western blotting (Fig. 6G-I).

## Discussion

In this study, PD-L1 levels were upregulated on peripheral neutrophils from patients with sepsis, which antagonized neutrophil apoptosis and was responsible for increased neutrophil accumulation in vital organs. The upregulation of neutrophil PD-L1 was found to be mediated by the glycolytic enzyme, PKM2. Specifically, under LPS stimulation, PKM2 translocated to the nucleus, directly interacted with the transcription factor STAT1, leading to its activation and increased expression of its target gene, PD-L1. In our previous study, upregulation of the glycolytic pathway was observed in primary neutrophils from patients with sepsis, and a regulatory role of LDHA-dependent glycolysis on neutrophil functions was identified [29]. Thus, the current findings signify a specific mechanism through which glycolysis may impact neutrophil physiology, involving the non-metabolic role of a glycolytic enzyme, PKM2, whose regulatory role on neutrophil activities during sepsis has not been explored previously. PKM2 regulated neutrophil PD-L1 expression and thereby affected neutrophil survival under LPS stimulation.

Under physiological conditions, circulating neutrophils are short-lived cells with a typical half-life of 8-20 h [40]. However, neutrophils remarkably extend their half-life during sepsis [41–44]. Both the intrinsic and extrinsic apoptotic pathways are altered in neutrophils under the stimulation of bacterial products, proinflammatory cytokines, and granulocyte–macrophage colony-stimulating factor [45]. Furthermore, multiple mechanisms were found to be involved, including activation of the NF-κB pathway [46, 47], elevation of the TNF-related apoptosis-inducing ligand (TRAIL) receptor 3 [48], suppression of caspases-3 and -9 [49], and downregulation of CD24, which normally activates the intrinsic apoptotic pathway [42]. This prolongation of neutrophil lifespan expands the circulatory neutrophil population and enhances neutrophil accumulation in organs [50], suggesting a potential impact of neutrophil apoptosis on the local inflammation of vital organs. In our in vitro experiments, PD-L1 blockade using a neutralizing antibody was able to restore neutrophil apoptosis under LPS stimulation. Alleviation of neutrophil organ accumulation was also observed in PD-L1^−/−^ mice subjected to sepsis induction, suggesting a potential modulatory role of PD-L1 on neutrophil retention in organs by regulating neutrophil apoptosis.

Our observation of upregulated PD-L1 levels on neutrophils during sepsis agreed with previous reports [51, 52], rendering the biological implication of neutrophil PD-L1 expression during sepsis worth exploring. It is well established that PD-L1^+^ neutrophils exert an immunosuppressive role by inhibiting the activation and survival of lymphocytes and monocytes through PD-1/PD-L1 interactions [26, 51]. Previous studies also identified intracellular motifs of PD-L1 capable of interacting with certain signaling pathways related to tumor initiation and progression, indicating that PD-L1 can exert intrinsic effects on PD-L1^+^ cells [53]. Regarding the intrinsic role of PD-L1 in neutrophils during sepsis, Patera et al*.* observed restoration of impaired neutrophil phagocytosis following the administration of anti-PD-L1 or anti-PD-1 antibodies to the whole blood of patients with sepsis [54]. Wang et al*.* reported impaired migratory capacity of PD-L1^+^ neutrophils compared with PD-L1^−^ neutrophils sorted from mice subjected to cecal ligation and puncture (CLP) [51]. In our in vitro experiments, delayed neutrophil apoptosis induced by LPS stimulation was reversed by the administration of a PD-L1 neutralizing antibody, which agrees with the findings of Wang et al. Through interference of primary human neutrophils using small-interfering RNA, they proposed a pro-survival role for PD-L1 on neutrophils by activating the PI3K/AKT pathway [51].It is also worth noticing that because neutrophils scarcely express PD-1 [55], this effect was probably independent of PD-1/PD-L1 ligation.

In keeping with our previous work identifying upregulated glycolytic genes including PKM2 and LDHA in LPS-activated primary human neutrophils [29], increased protein levels of PKM2 in peripheral neutrophils from patients with sepsis and LPS-stimulated dHL-60 cells was observed. As one of the four isomers of pyruvate kinase, PKM2 exists in both tetrameric and dimeric forms. The tetrameric form mainly serves an enzymatic role in the cytoplasm, converting phosphoenolpyruvate to pyruvate. The dimeric form has low catalytic activity but can undergo nuclear translocation depending on its C-terminal nuclear localization signal [56]. In the nucleus, the PKM2 dimer can interact with proteins including β-catenin, Hif-1α, and NF-κB and serve as a protein kinase. This functional property of PKM2 dimer makes it able to modulate gene transcription [57–60]. In terms of immunoregulation, PKM2 nuclear translocation is implicated in T cell differentiation and activation during autoimmune encephalopathy [61, 62]. It also promotes macrophage-mediated inflammation in sepsis by promoting metabolic reprogramming and acting as a cofactor of Hif-1α [63]. More recently, Dhanesha et al*.* found that PKM2 activation of STAT3-mediated neutrophil hyperinflammation during ischemic stroke [64]. The impact of PKM2 on neutrophil function during sepsis has not been explored. Thus, our study contributes to this field of knowledge by identifying the involvement of PKM2 in sepsis-induced upregulation of PD-L1 expression on neutrophils. Reversal of PD-L1 upregulation on neutrophils was observed under pharmacological intervention of PKM2 activity both in vivo and in vitro. The specific mechanism involving PKM2 nuclear translocation and STAT1 activation was also demonstrated by our in vitro experiments.

Our findings expand on the knowledge of the regulatory mechanisms of neutrophil PD-L1 expression under septic conditions, which remain underexplored with only two studies highlighting the role of spleen-derived IFN-γ [65, 66] and another highlighting the involvement of the p38α-MSK1/MK2 pathway in the generation of PD-L1^+^ neutrophils during sepsis [26]. Our study stresses the non-metabolic role of PKM2, which is also reminiscent of the regulatory role of tumor-derived lactate on neutrophil PD-L1 expression. This also occurs in a non-metabolic manner through activation of the NF-κB and COX2-PGE2 pathway [27]. With more observations on the direct involvement of glycolytic enzymes and intermediaries in intracellular signaling and gene-expression regulation, the regulatory capacity of PKM2 on other aspects of neutrophil activities under septic conditions has considerable research potential.

Enhanced organ accumulation of neutrophils may improve pathogen clearance, but there is also a possibility of impeded inflammation resolution and tissue injury [50, 67]. Our current findings lead to speculation about whether the induction of neutrophil apoptosis can serve a tissue-protective role and whether PD-L1 or its regulator PKM2 can be targeted to alleviate organ injury during sepsis. Several agents with pro-apoptotic effects on neutrophils, including TRAIL, the selective Mer inhibitor, UNC2250, and doxorubicin delivered by nanoparticles were reported to have therapeutic potential because they alleviate neutrophil retention in organs during sepsis [68–70], suggesting the induction of neutrophil apoptosis could be a therapeutic option for this condition. However, attention should be paid to the timing of intervention since it has been shown that eliminating neutrophils prior to the onset of sepsis augments bacteremia [71]. Meanwhile, although the association between PD-L1 expression and neutrophil longevity is established, previous studies have mixed conclusions regarding the impact of PD-L1 expression on organ damage. This may be attributed to a focus on different cell targets for PD-L1. For example, in acute lung injury during sepsis, PD-L1 aggravats lung injury in CLP models by disturbing the stability of lung vascular endothelial cells, as shown in several studies [72–74]. In contrast, PD-L1 is also shown to limit lung tissue inflammation by alleviating T cell cytokine production and promoting the anti-inflammatory role of Treg cells [75, 76]. Using different models of acute lung injury (i.e., direct versus indirect lung injury) may also cause inconsistent results. The role of PD-L1 on sepsis-induced liver injury remains less explored, with one study identifying a therapeutic role for anti-PD-L1 antibodies on liver damage in a CLP model [77] and another demonstrating that PD-L1 can cause liver sinusoidal endothelial cell damage by interacting with PD-1 on Kupffer cells [78]. Our study only focused on the impact of PD-L1 on the amount of neutrophils that accumulat in vital organs during sepsis. Further exploration is needed to gain more insight into the exact impact of this phenomenon on damage to any specific organ and its potential to be applied to the development of novel therapies.

## Conclusions

The current study demonstrates that the non-metabolic function of the glycolytic enzyme, PKM2, is involved in PD-L1 upregulation on septic neutrophils, which exerts an anti-apoptotic effect on neutrophils and impacts neutrophil infiltration in vital organs. The findings suggest that PKM2 and PD-L1 could serve as potential therapeutic targets for sepsis. In addition, our study suggests a need for further investigations into how metabolic reprogramming may shape the neutrophil phenotype during sepsis through metabolic-dependent or -independent mechanisms.

## Materials and methods

### Patients and blood sample processing

Adult patients meeting the Sepsis 3.0 criteria [79] were recruited from the Department of Critical Care Medicine, Ruijin Hospital, Shanghai Jiaotong University School of Medicine. Healthy adult volunteers were recruited as control subjects. The study protocols were approved by the Human Research Ethics Committee of Ruijin Hospital. Informed consent was obtained from all patients or their legally-authorized representatives. The information collected included baseline patient demographics, infection site, acute physiology and chronic health evaluation (APACHE) II score, sequential organ failure assessment (SOFA) score, length of stay in the intensive care unit (ICU), and outcome (survival or death in the ICU).

Within 24 h after sepsis diagnosis, 12 ml of venous blood was collected from the patients using ethylenediaminetetraacetic acid (EDTA) anti-coagulation tubes. For the healthy volunteers, 25 ml of venous blood was drawn. Blood samples were stored at 4℃ and processed within 4 h. Peripheral blood neutrophils were isolated through density-gradient centrifugation using Histopaque 11191 and 10771 (Sigma-Aldrich, St. Louis, MO, USA) as previously described [80]. The purity of isolated neutrophils was > 95% as assessed by flowcytometry (Supplementary Fig. 1). After isolation, neutrophils were resuspended in phosphate-buffered saline (PBS) at a density of 2 × 10^6^ cells/ml and subjected to immediate flow cytometric staining or protein extraction processes.

### Cell culture and differentiation

The human promyeoloblast cell line, HL-60, was purchased from the Cell Bank of the Chinese Academy of Sciences, Shanghai. Cells were cultured in Roswell Park Memorial Institute (RPMI) 1640 medium supplemented with 15% fetal bovine serum and 1% penicillin and streptomycin (Gibco, Grand Island, NY, USA) in a humidified incubator set at 37 °C with 5% CO_2_. The cells were passed every 2 days with the cell density maintained between 5 × 10^5^ to 2 × 10^6^ cells/ml. To differentiate HL-60 cells into neutrophil-like cells, the cells were cultured in fresh complete medium supplemented with 1.3% (v/v) dimethyl sulfoxide (DMSO) (Sangon Biotech, Shanghai, China) for 5 days.

### Animal procedures

Wild type and PD-L1^−/−^ C57BL/6 J mice (male, 8–12 weeks old) were purchased from Shanghai Model Organisms (Shanghai, China) and kept at room temperature under a constant 12 h light/dark cycle. Mice were housed five per cage with access to food and water ad libitum. Mice were randomly assigned to either the experimental or control groups. All experiments were performed according to protocols approved by the University Committee for Laboratory Animals and adhered to the guidelines of the Shanghai Institutes for Biological Sciences Council on Animal Care.

The sepsis animal model was established by intraperitoneal injection of lipopolysaccharide (LPS) (5 mg/kg) dissolved in 100 μl PBS. Mice injected with an equal volume of PBS served as controls. After 16 h, the mice were sacrificed to harvest the lungs, livers, or llower limbs. The left lung and liver of each mouse were fixed in 4% formaldehyde (Servicebio, Wuhan, Hubei, China) for histological analysis, while the right lung was processed for flow cytometric analysis. After careful removal of muscles from the lower extremities, the tibias and femurs were collected, immersed in ice-cold, complete RPMI 1640 medium, and prepared for the collection of bone marrow cells.

### Flow cytometry

PE anti-human CD274 (B7-H1, PD-L1), PE anti-mouse CD274 (B7-H1, PD-L1), APC anti-mouse/human CD11b, Brilliant Violet 421™ anti-mouse Ly6G, and FITC anti-mouse CD45 antibodies, as well as the Zombie NIR™ Fixable Viability Kit were purchased from Biolegend (San Diego, CA, USA). The Annexin V-FITC/propidium iodide (annexin V-FITC/PI) Cell Apoptosis Detection Kit was purchased from Yeasen (Shanghai, China). Before staining with antibodies against the target molecules, cells were incubated with Fc receptor blockers (Biolegend, San Diego, CA, USA) for 15 min at room temperature.

To determine the surface PD-L1 levels of isolated peripheral neutrophils and dHL-60s, cells were resuspended in PBS and stained with antibodies against human/mouse PD-L1 for 25 min at 4 °C. To detect apoptotic dHL-60s, cells were resuspended in 1 × binding buffer and stained with a mixture of annexin V-FITC and PI for 15 min at room temperature.

To determine the surface PD-L1 level of bone marrow neutrophils, epiphyses were cut off and bone marrow cells were flashed out from both ends of the tibia/femur using a 25-gauge needle and a 12-cc syringe filled with RPMI complete medium supplemented with 2 mM EDTA. The suspension was filtered through a 70-μm filter and red blood cell lysis was performed. Cells were resuspended in PBS and stained with antibodies against CD11b, Ly6G, and PD-L1 for 25 min at 4 °C.

To determine the number of neutrophils in the lungs, the right lung lobe was manually dissociated with scissors and incubated with 1.5 mg/ml collagenase A and 0.1 mg/ml DNase at 37 °C for 45 min. Next, the mixture was mashed through a 70 μm sieve to generate single-cell suspensions and red blood cell lysis was performed. Cells were resuspended in 200 μl PBS and stained with dead/live stain for 20 min at room temperature before staining with antibodies against CD45, CD11b, and Ly6G for 25 min at 4 °C.

After the staining processes, cells were washed twice and analyzed on a BD FACSLyric flow cytometer (BD Biosciences, Franklin Lakes, NJ, USA). Data analysis was performed using FlowJo (Tree Star, Ashland, OR, USA).

### Western blotting

Monoclonal antibodies against PKM2, STAT1, p-STAT1, pro-caspase-3, cleaved caspase-3, and Mcl-1 were purchased from Cell Signaling Technology (Boston, MA, USA).

Cells were collected, washed with PBS and placed into 1 × Radio-Immunoprecipitation Assay (RIPA) buffer (Thermo Fisher Scientific, Waltham, MA, USA). After lysing the cells for 45 min on ice, supernatants were collected through centrifugation at 16,000 × *g*, 4 °C for 10 min and boiled for 10 min with loading buffer (Epienzyme, Shanghai, China). Total protein (25 μg), as measured by the bicinchoninic acid method, was separated on 10% polyacrylamide gels and transferred onto polyvinylidene difluoride (PVDF) membranes of 0.45 µm pore size. The membranes were blocked and incubated with primary antibodies diluted at a ratio of 1:1000 overnight at 4 °C. Next, the membranes were washed with 1 × Tris-buffered saline with Tween 20 (TBST) 3 times and incubated with horseradish peroxidase (HRP)-linked secondary antibodies for 1 h at room temperature. Specific bands were observed by enhanced chemiluminescence (ECL).

### Co-immunoprecipitation

Co-immunoprecipitation was performed using the IP/CoIP kit (Absin, Shanghai, China) according to the manufacturer’s instructions. Primary antibodies against PKM2 and STAT1 were purchased from Cell Signaling Technology (Boston, MA, USA) and Abcam (Cambridge, CB2 0AX, UK), respectively. The IgG isotype control was purchased from Cell Signaling Technology (Boston, MA, USA). A secondary antibody specific to the IgG light chain was purchased from ABclonal (Wuhan, China).

### Immunohistochemistry

After fixation, tissues were paraffin-embedded and cut into 4 μm sections. The sections then underwent xylene deparaffinization, rehydration with 30–100% ethanol, and boiling in retrieval buffer (10 mM citrate buffer, pH 6.0) for 5 min. Tissue sections were blocked with 5% bovine serum albumin and incubated with an anti-Ly6G antibody (1:100) (Cell Signaling Technology, Boston, MA, USA) at 4 °C overnight. Afterward, the sections were then incubated with a secondary antibody (1:200) at room temperature for 30 min. After staining with 3,3'-diaminobenzidine (Sigma-Aldrich, Saint Louis, MO, USA) and counterstaining with hematoxylin and eosin (H&E) (Yeasen, Shanghai, China), the tissue sections were scanned with NanoZoomer S360 (Hamamatsu Photonics, Naka-ku Hamamatsu City, Shizuoka Pref., Japan).

### Statistical analysis

Data analyses were performed with GraphPad (version 7.0.0; GraphPad Software Inc., La Jolla, CA, USA). Data are shown as means ± SEM for at least three independent experiments. Data from two groups with nonnormal distribution were compared using the Mann–Whitney U test. Data from multiple groups were compared using one-way ANOVA. *P* < 0.05 was considered a statistically significant difference.

## Supplementary Information

**Additional file 1: Supplementary Figure 1.** Purity of isolated peripheral blood neutrophils, verified by measurement of CD11b, CD16 and CD66b expression through cytometry. **Supplementary Figure 2A.** Gating strategy for human peripheral blood neutrophils. **Supplementary Figure 2B.** Gating strategy for dHL-60s.

**Additional file 2.**

## Data Availability

All data generated or analyzed during this study are included in this article.

## Abbreviations

PD-L1: Programmed death ligand-1
PKM2: Pyruvate kinase M2
DMSO: Dimethyl sulfoxide
LPS: Lipopolysaccharide
Annexin V/PI: Annexin V/propidium iodide
STAT1: Signal transducer and activator of transcription 1
PD-1: Programmed death-1
PD-L: Programmed death ligand
HK2: Hexokinase 2
GAPDH: Glyceraldehyde-3-phosphate dehydrogenase
LDHA: Lactate dehydrogenase A
MFI: Mean fluorescence intensity
dHL-60s: DMSO-differentiated HL-60s
TRAIL: TNF-related apoptosis inducing ligand
CLP: Cecal ligation and puncture
APACHE II: Acute physiology and chronic health evaluation II
SOFA: Sequential organ failure assessment
ICU: Intensive care unit
EDTA: Ethylenediaminetetraacetic acid
PBS: Phosphate buffered saline
RPMI: Roswell Park Memorial Institute
RIPA: Radio-immunoprecipitation assay
PVDF: Polyvinylidene difluoride
TBST: Tris buffered saline with Tween 20
HRP: Horseradish peroxidase
ECL: Enhanced chemiluminescence
H&E: Hematoxylin and eosin
