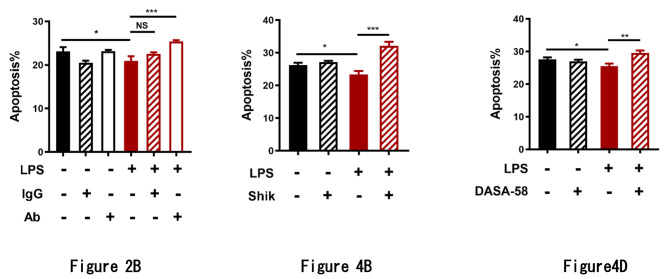# Correction: PKM2/STAT1-mediated PD-L1 upregulation on neutrophils during sepsis promotes neutrophil organ accumulation by serving an anti-apoptotic role

**DOI:** 10.1186/s12950-024-00406-w

**Published:** 2024-09-04

**Authors:** Yinjiaozhi Li, Ruoming Tan, Ranran Li, Rui Tian, Zhaojun Liu, Xiaoli Wang, Erzhen Chen, Tingting Pan, Hongping Qu

**Affiliations:** 1 Department of Critical Care Medicine, Ruijin Hospital, Shanghai Jiao Tong University School of Medicine, 197 Ruijin Er Road, Shanghai, 200025 China; 2 Department of Emergency Medicine, Ruijin Hospital, Shanghai Jiao Tong University School of Medicine, 197 Ruijin Er Road, Shanghai, 200025 China

**Correction to: Li et al. Journal of Inflammation (2023) 20:16**

10.1186/s12950-023-00341-2

The original version of this publication contained errors in Figs. 2B and 4B, and 4D. Those figures represented the results of the apoptosis measurement by flowcytometry, where both the Q2 population (Annexin V+/PI + cells) and the Q3 population (Annexin V+/PI- cells) were counted as apoptotic cells.

However, the Q2 population also contained necrotic cells which should not be included in apoptotic cells and only the Q3 population should be regarded as truly apoptotic cells.

Regarding this concern, the authors explained that although their interpretation of the flowcytometry data may be inaccurate, the conclusions were not affected, and their conclusions were also supported by the Western blot results. The editors agree with the authors’ response based on the following notions:

Firstly, the early apoptotic cells (LR quadrant) show the same patterns of increase/decrease as the overall aV + population, and the authors’ additional analysis of aV+/PI- cells confirms that this does not affect the outcome.

Secondly, the data are combined with western blot analysis of both pro- and antiapoptotic proteins, which further supports their conclusions.

Therefore, we provide a correction to update the bar graphs in Figs. 2B and 4B, and 4D as shown below:

The original bar graphs:

**Figure 1:**
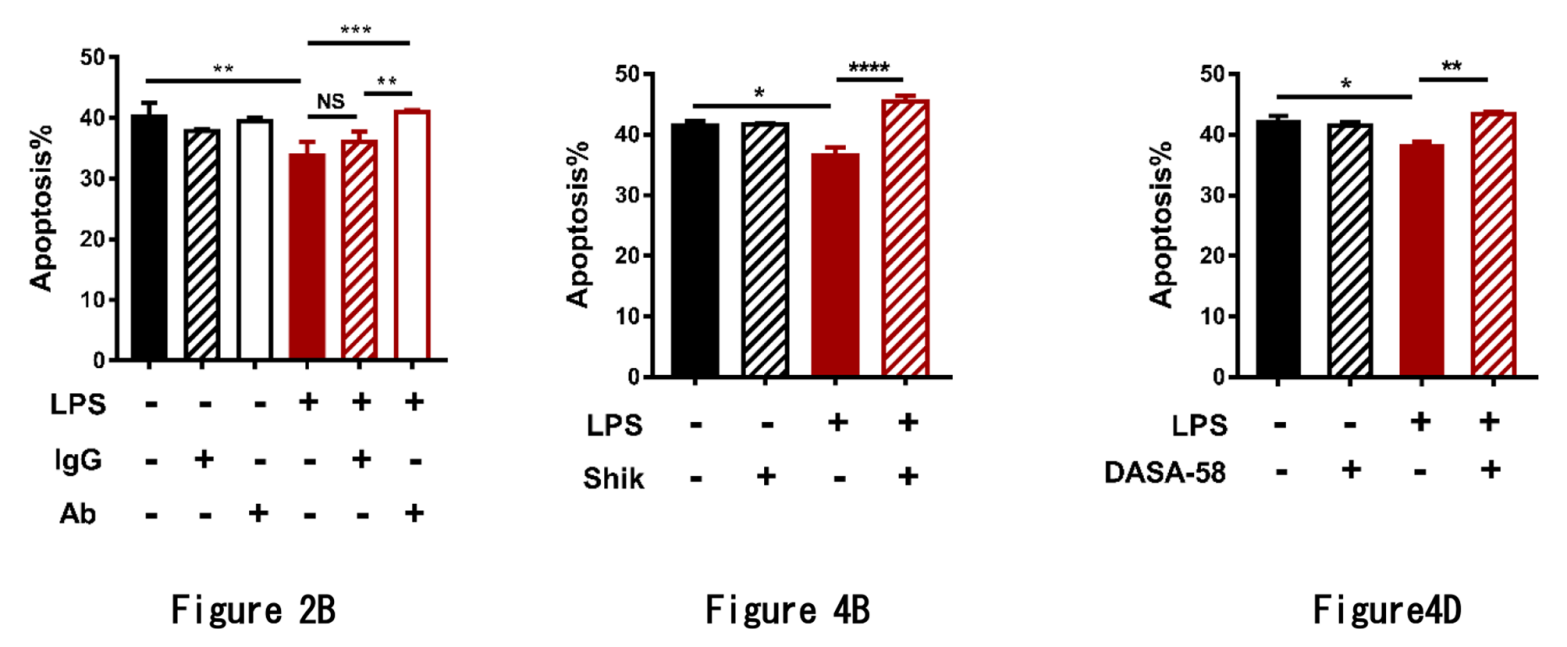

The updated bar graphs:

**Figure:**