# Supplementary material for: PKM2/STAT1-mediated PD-L1 upregulation on neutrophils during sepsis promotes neutrophil organ accumulation by serving an anti-apoptotic role
Source: J Inflamm (Lond). 2023 May 2;20:16. doi: 10.1186/s12950-023-00341-2 (PMC10155438; doi:10.1186/s12950-023-00341-2)
Supplement: Supplementary file 1 — Additional file 1: Supplementary Figure 1. Purity of isolated peripheral blood neutrophils, verified by measurement of CD11b, CD16 and CD66b expression through cytometry. Supplementary Figure 2A. Gating strategy for human peripheral blood neutrophils. Supplementary Figure 2B. Gating strategy for dHL-60s. [file 12950_2023_341_MOESM1_ESM.docx]

**Supplementary Figures**

**Supplementary Figure 1**


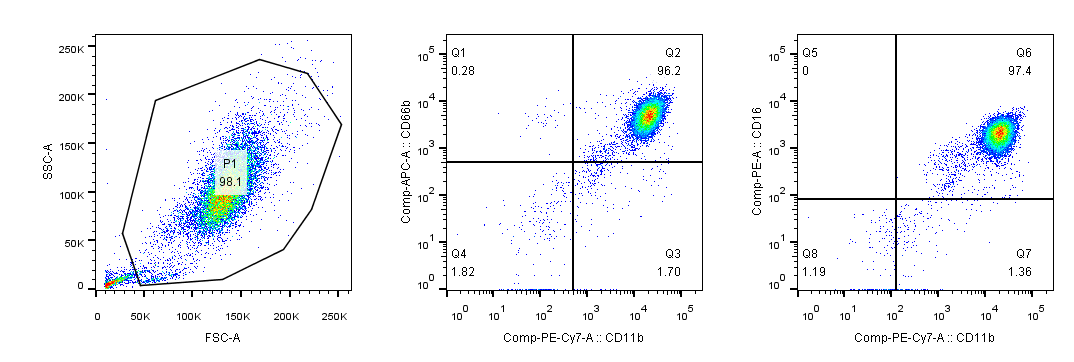


Supplementary Figure 1, Purity of isolated peripheral blood neutrophils, verified by measurement of CD11b, CD16 and CD66b expression through cytometry.

**Supplementary Figure 2**


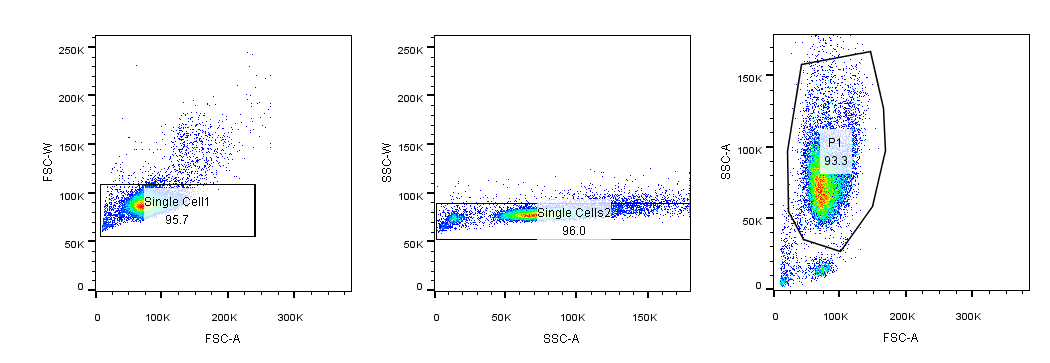


Supplementary Figure 2A Gating strategy for human peripheral blood neutrophils.


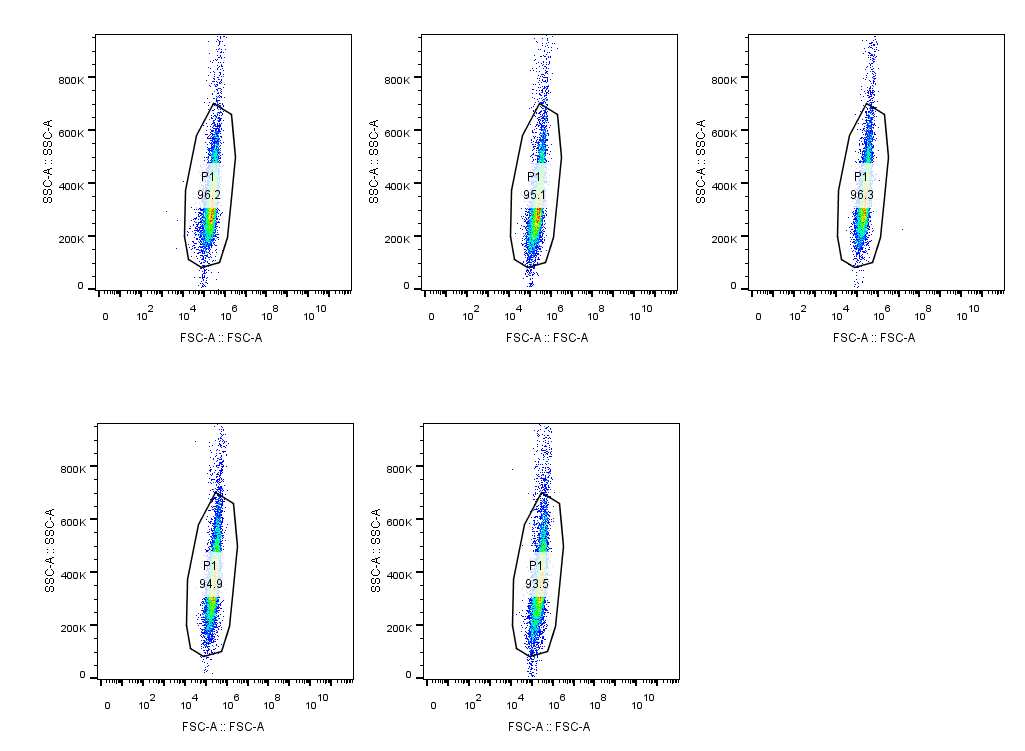


Supplementary Figure 2B Gating strategy for dHL-60s.
