# Supplementary material for: PKM2/STAT1-mediated PD-L1 upregulation on neutrophils during sepsis promotes neutrophil organ accumulation by serving an anti-apoptotic role
Source: J Inflamm (Lond). 2023 May 2;20:16. doi: 10.1186/s12950-023-00341-2 (PMC10155438; doi:10.1186/s12950-023-00341-2)
Supplement: Supplementary file 2 — Additional file 2. [file 12950_2023_341_MOESM2_ESM.docx]

**Figure 2C**

Pro caspase3


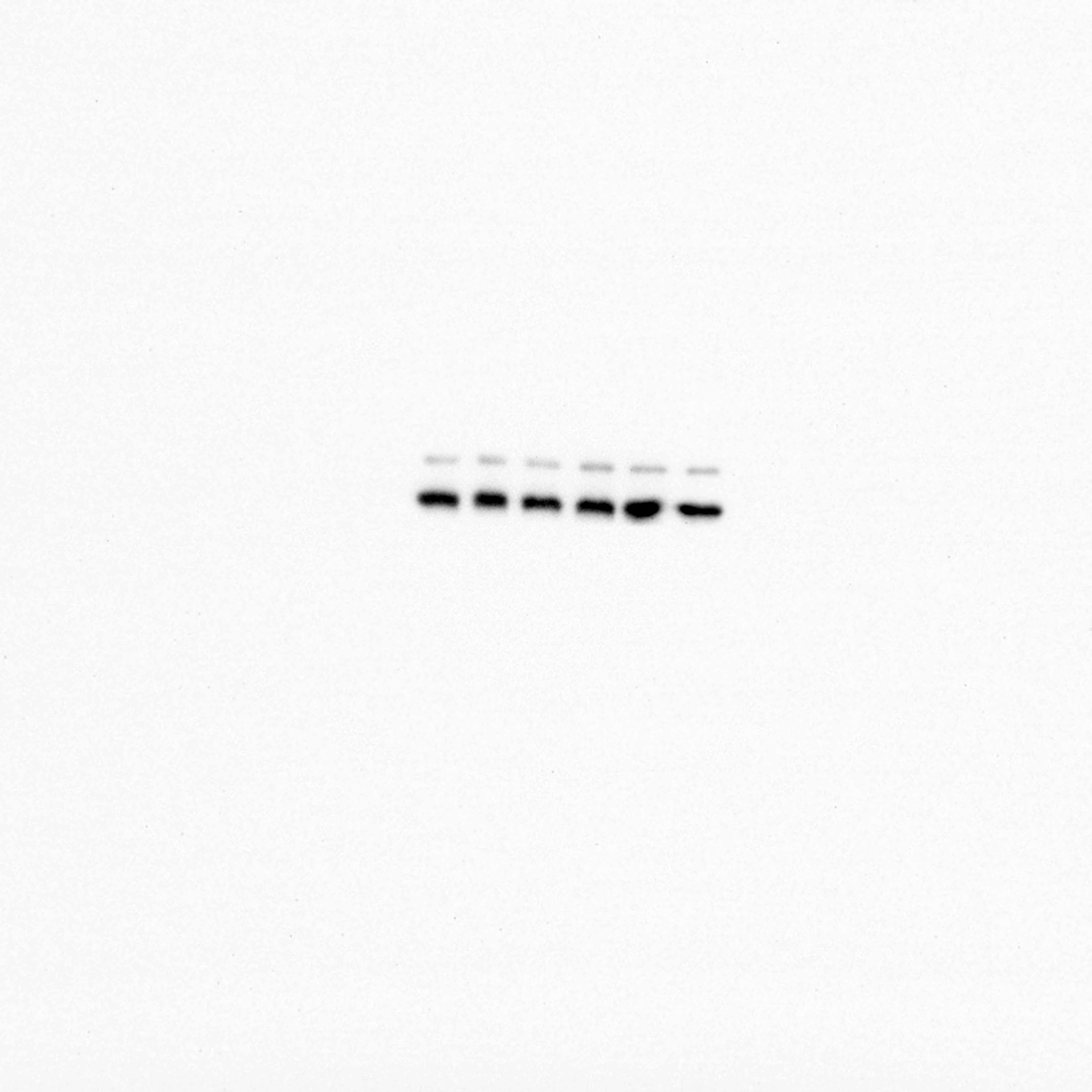


**35kD**

Cleaved caspase3


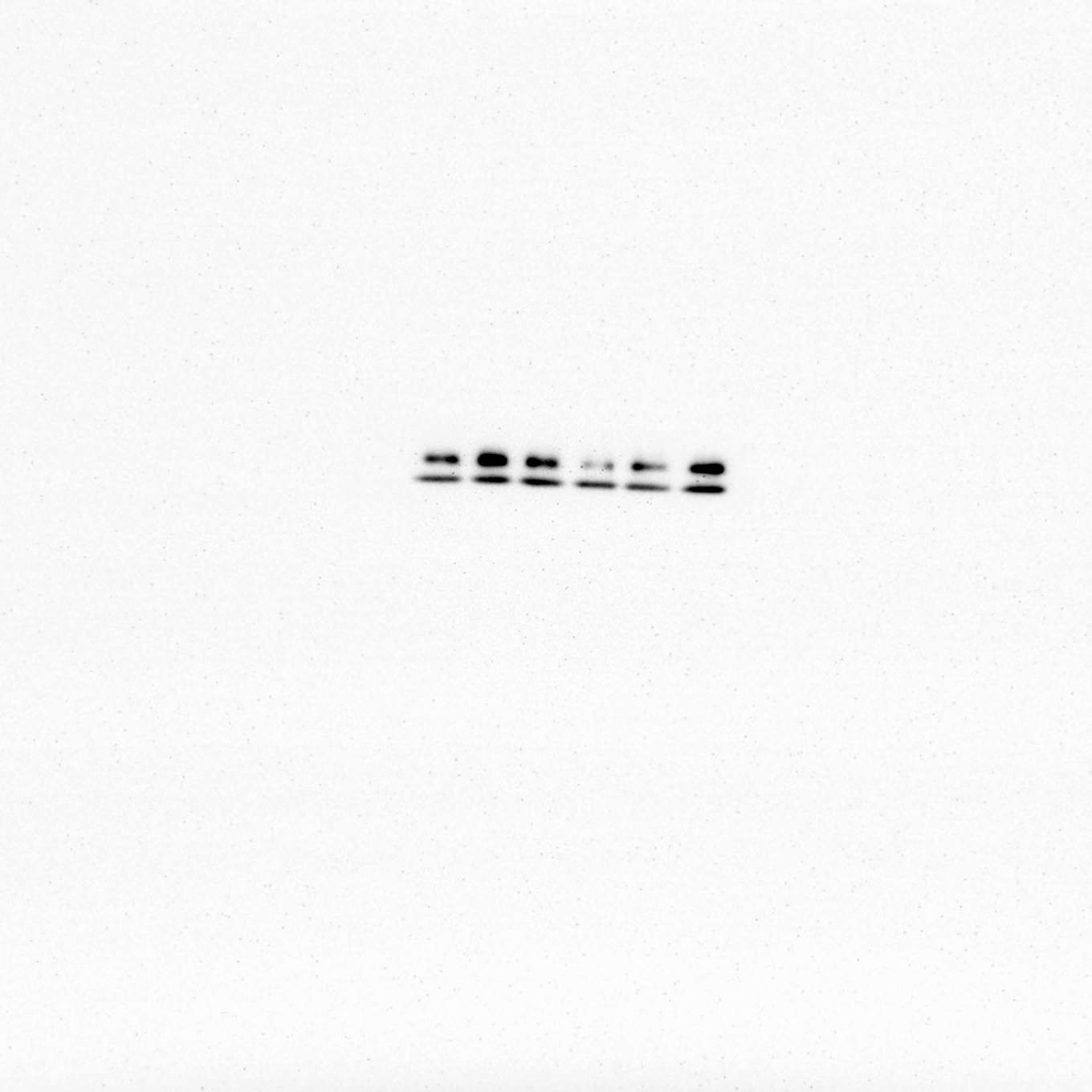


**15kD**

Mcl-1


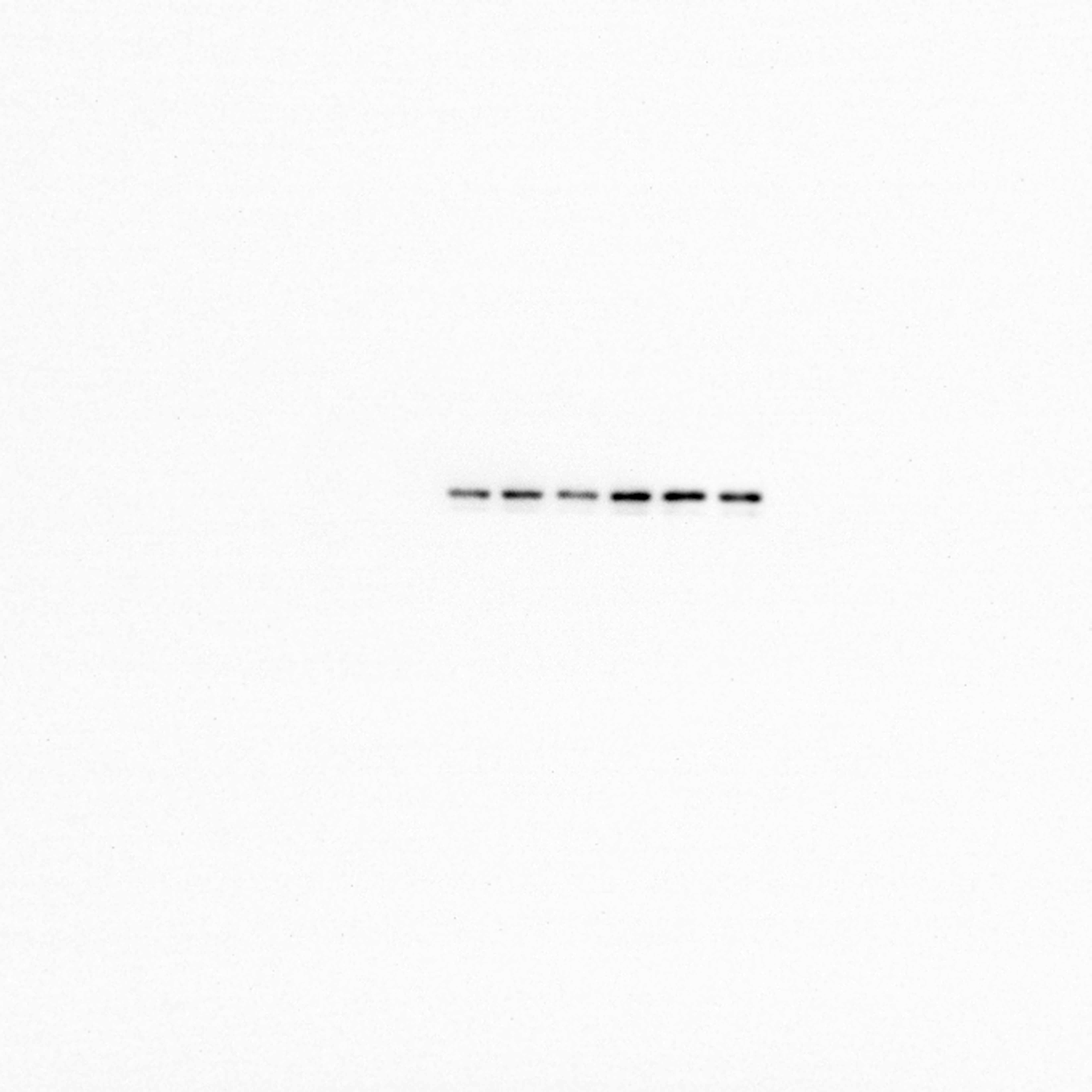


**40kD**

Hsp90


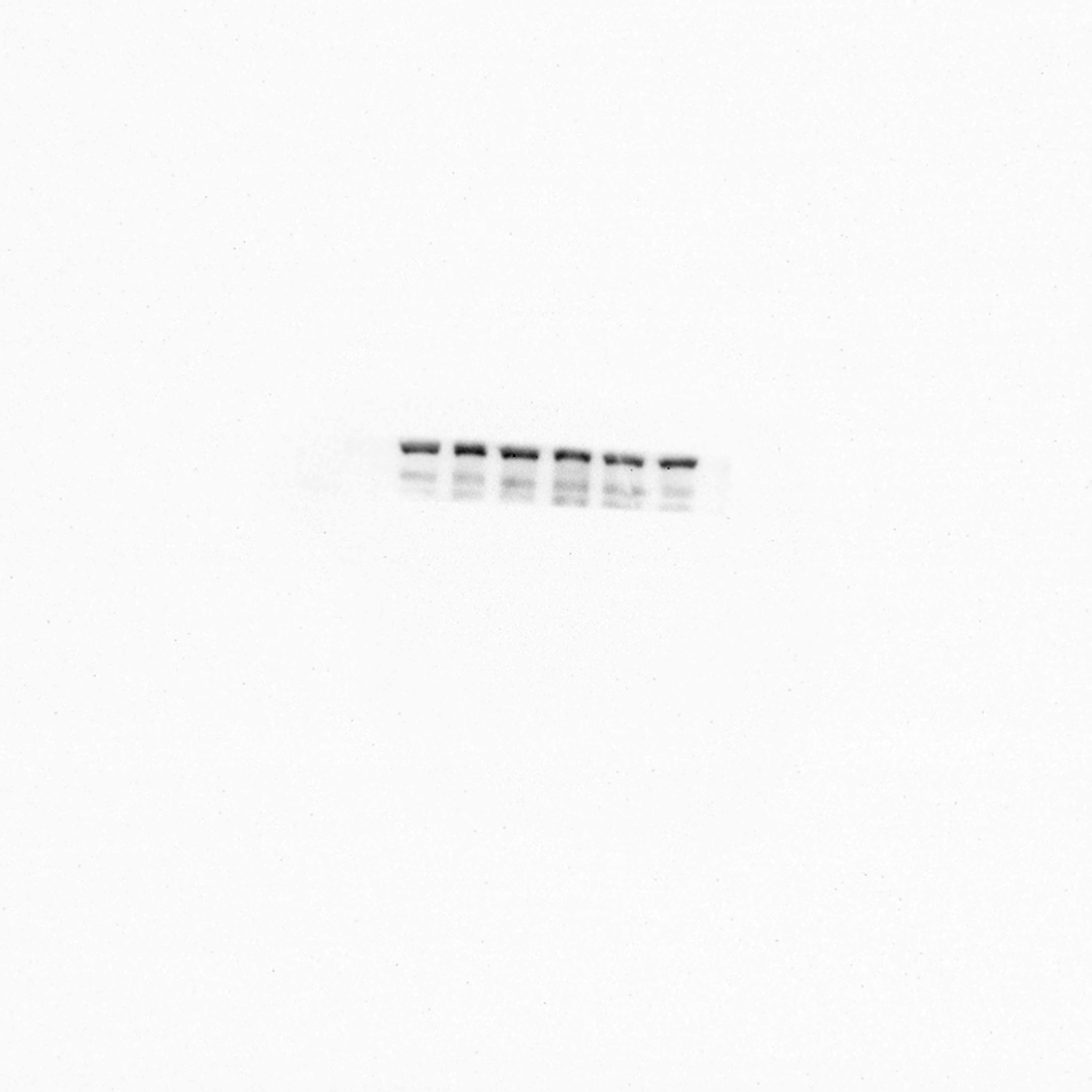


**90kD**

**Figure 3A**

PKM2


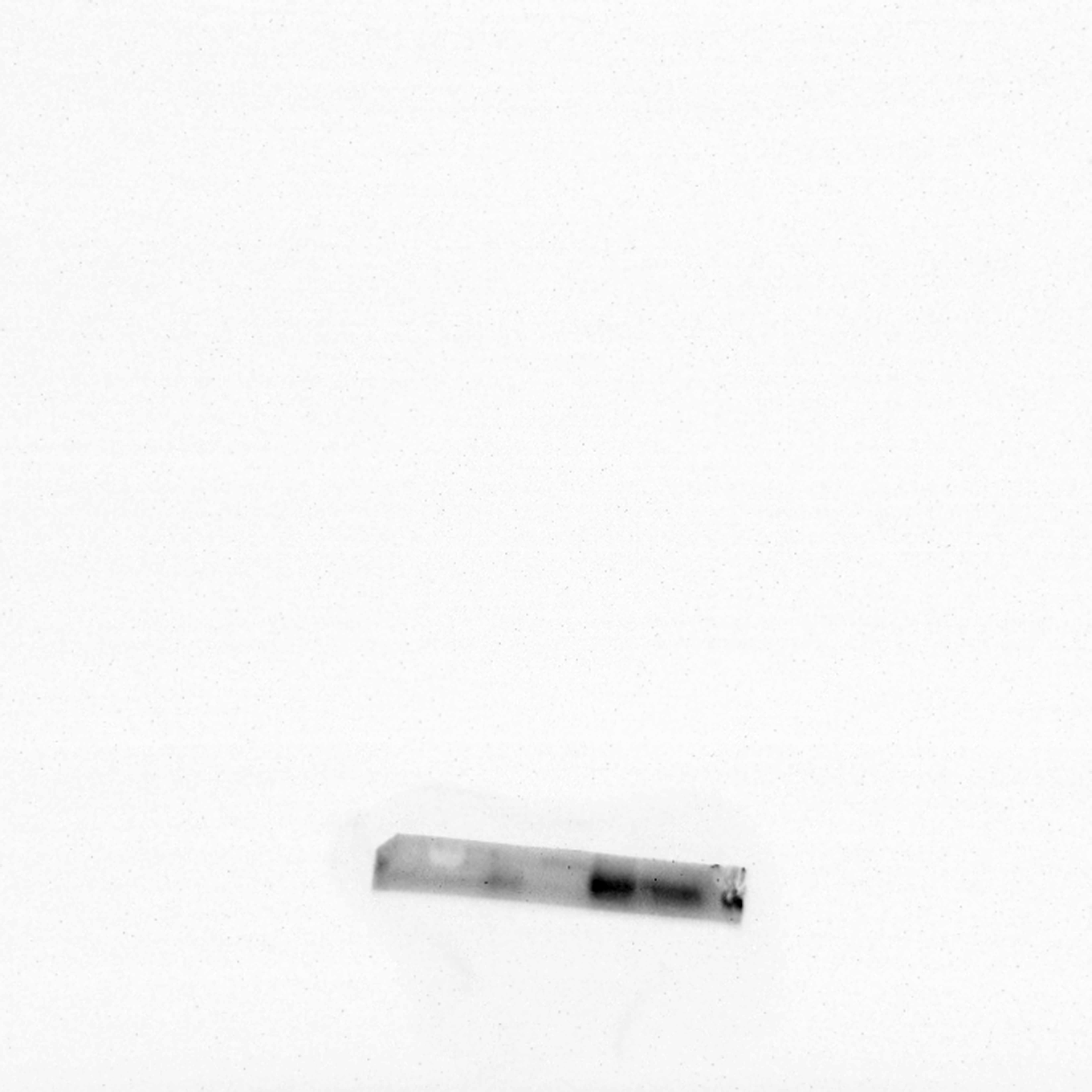


**60kD**

Actin


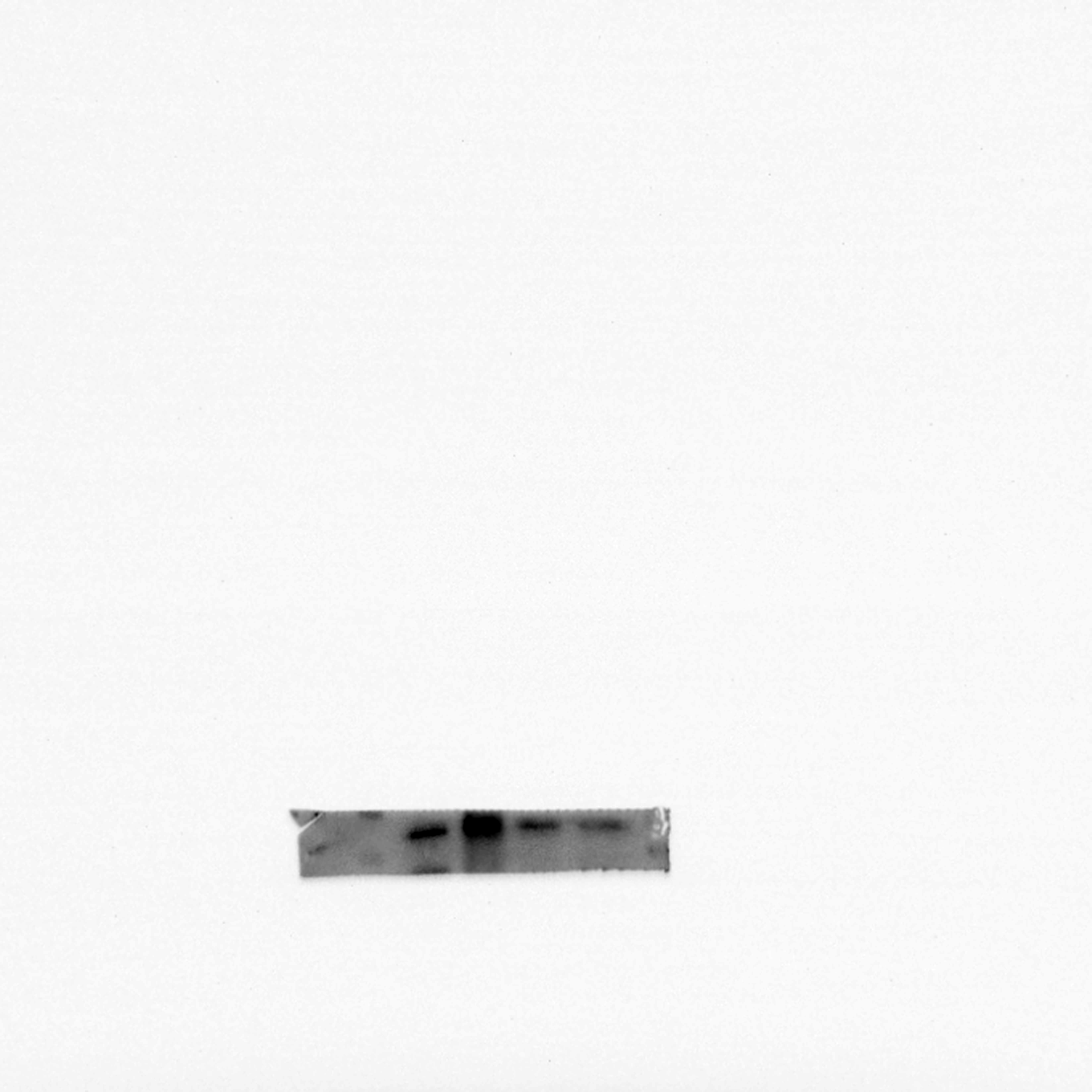


**42kD**

**Figure 3D**

PKM2


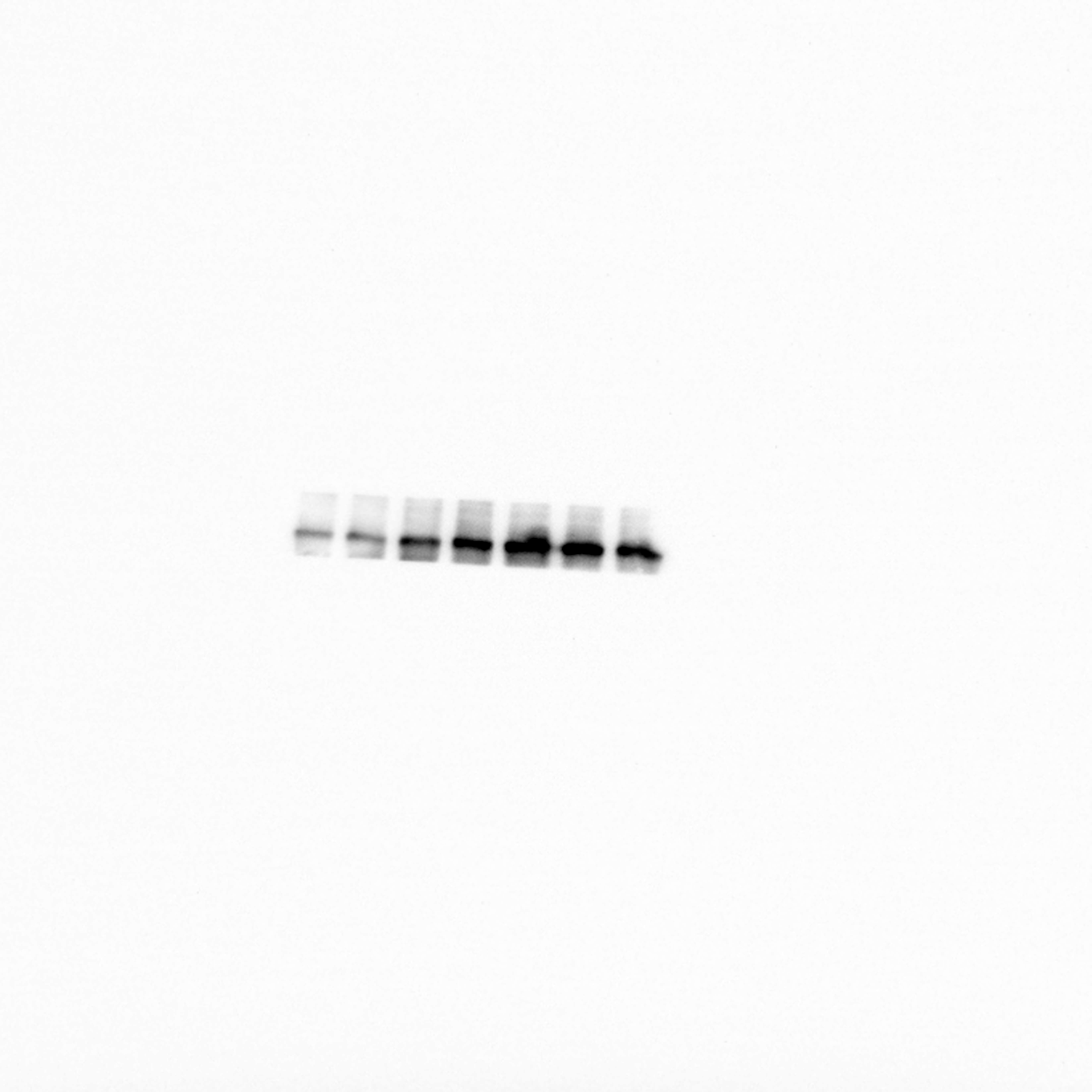


**60kD**

Actin


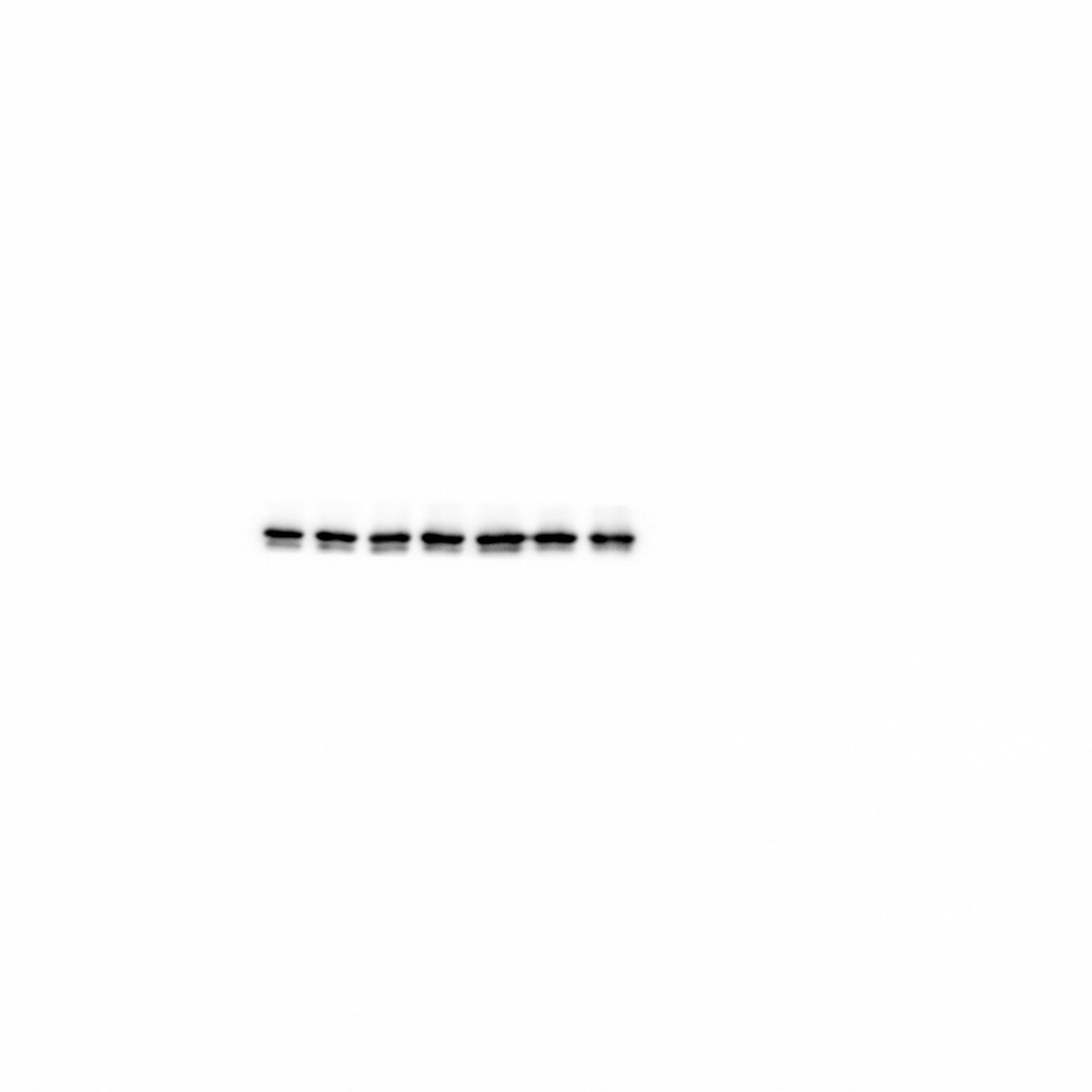


**42kD**

**Figure 4E**

Pro caspase3


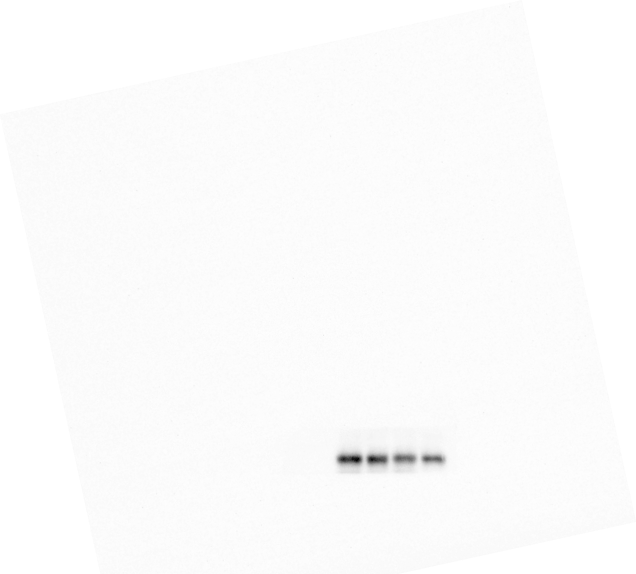


**35kD**

Cleaved caspase3


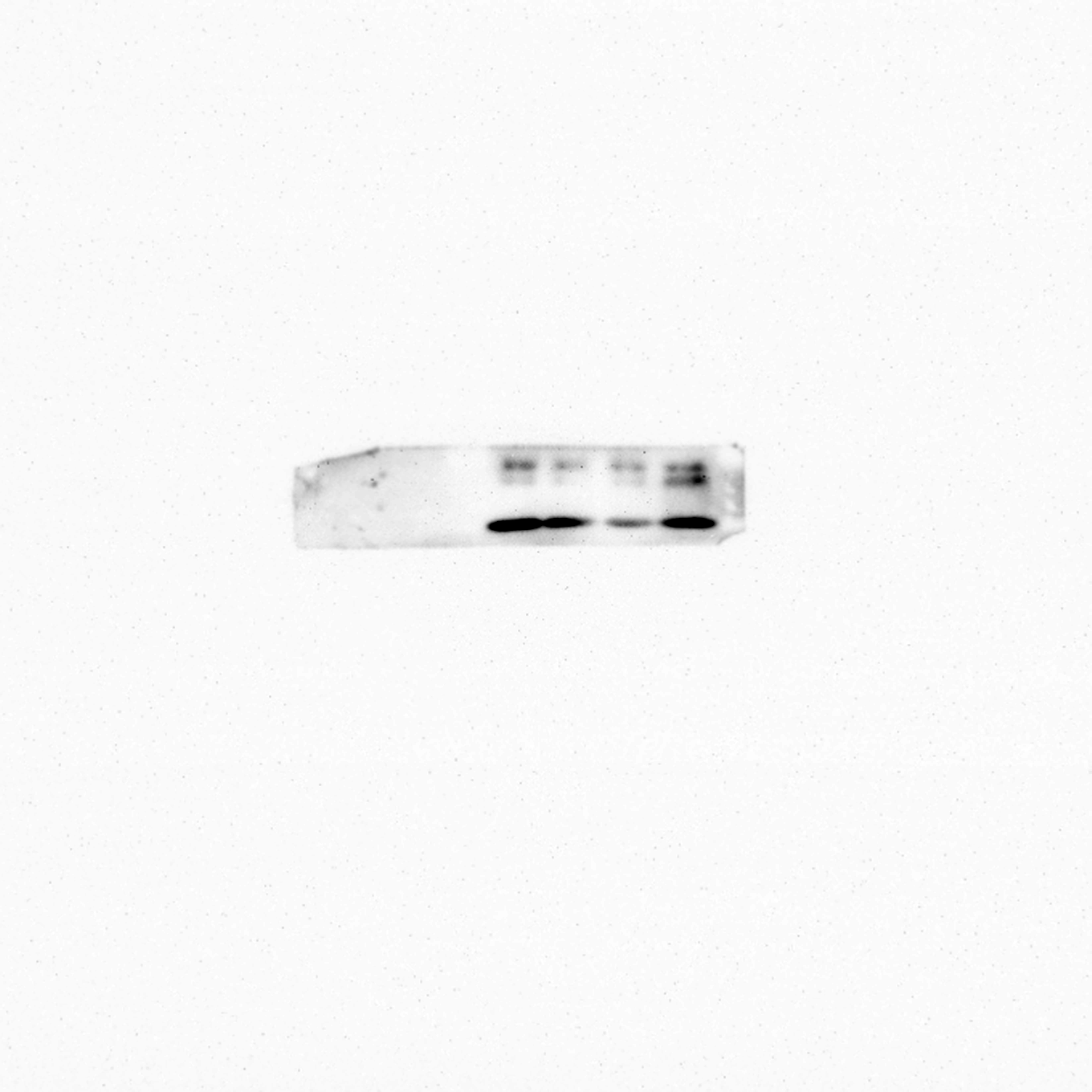


**15kD**

Mcl-1


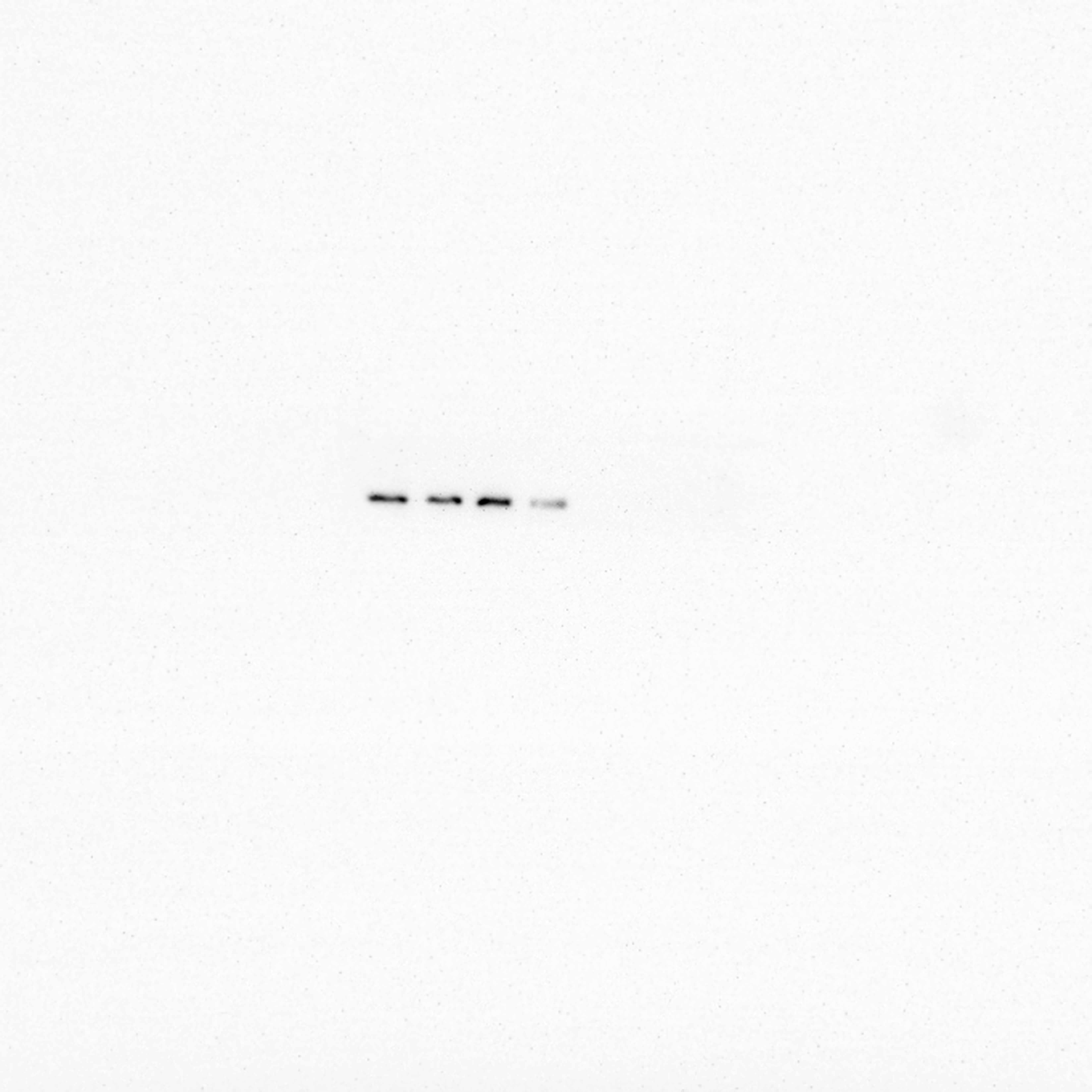


**40kD**

Hsp90


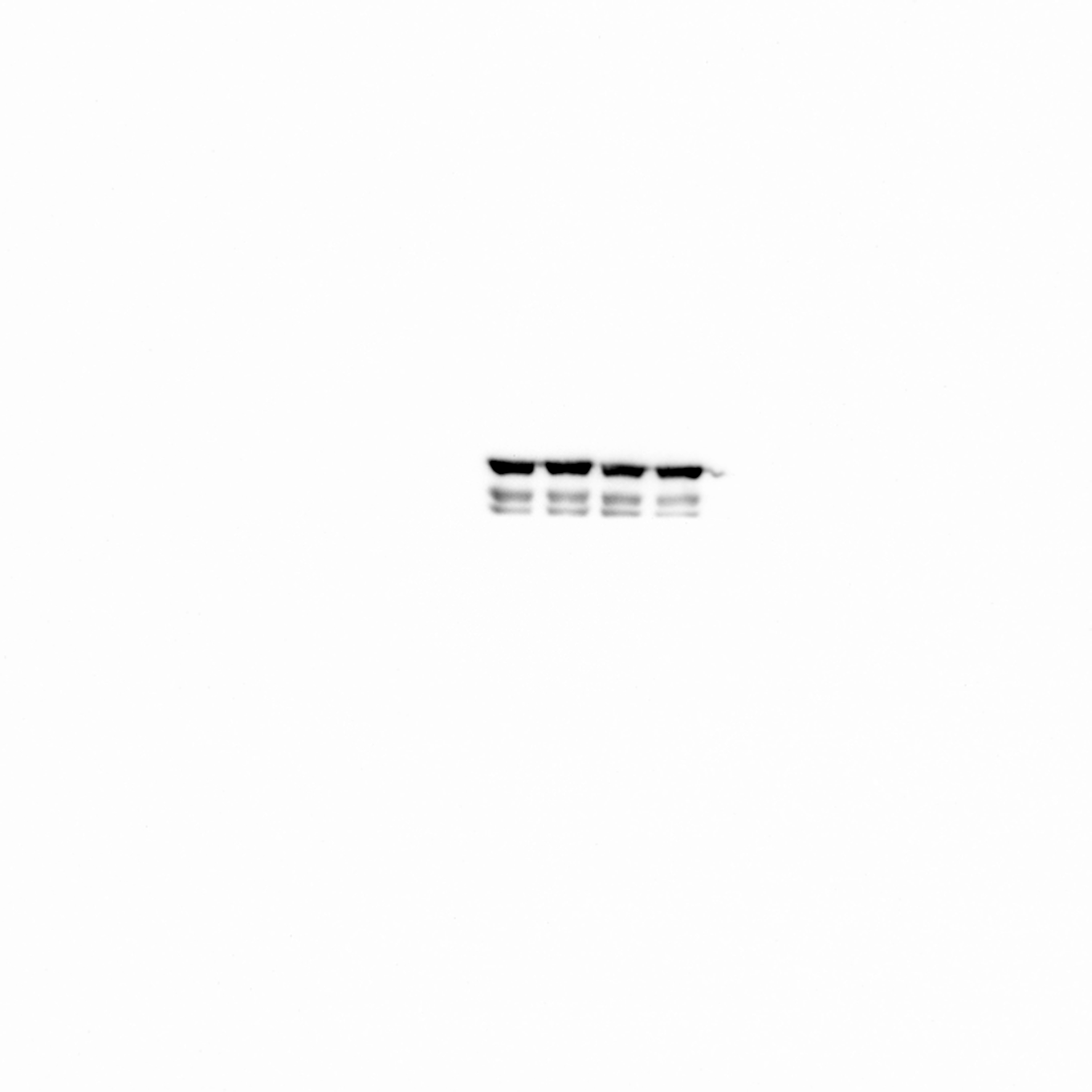


**90kD**

**Figure 4H**

Pro caspase3


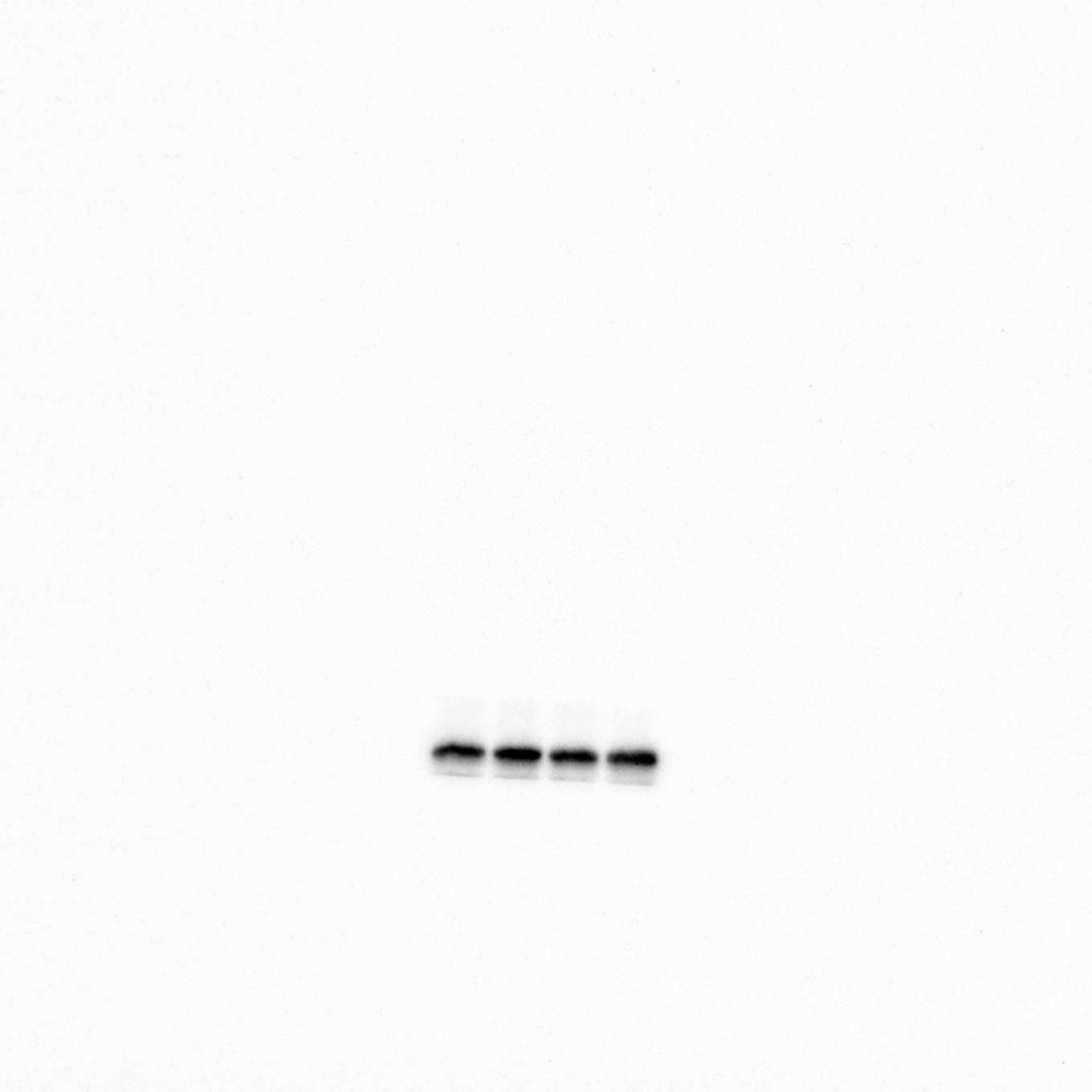


**35kD**

Cleaved caspase3


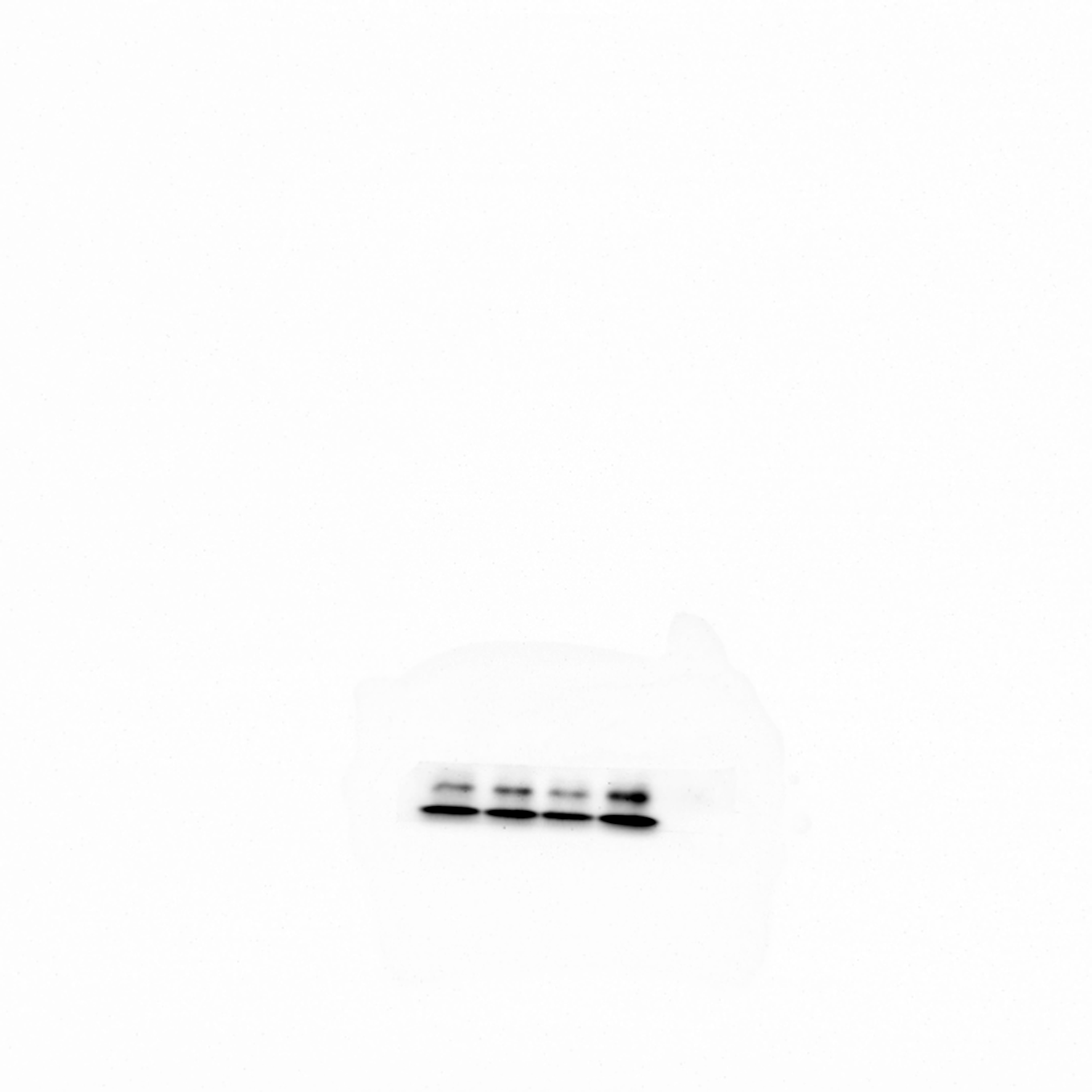


**15kD**

Mcl-1


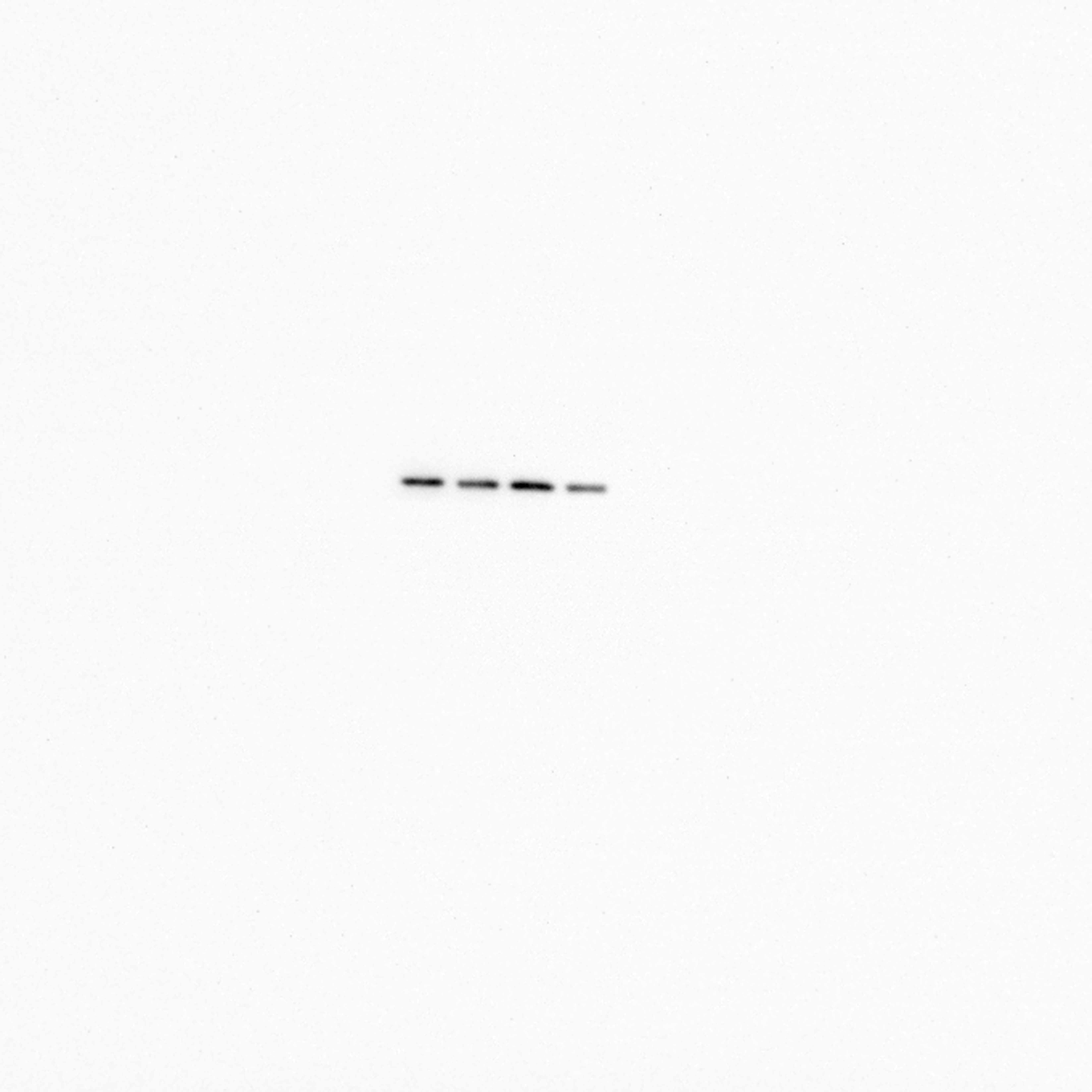


**40kD**

Hsp90


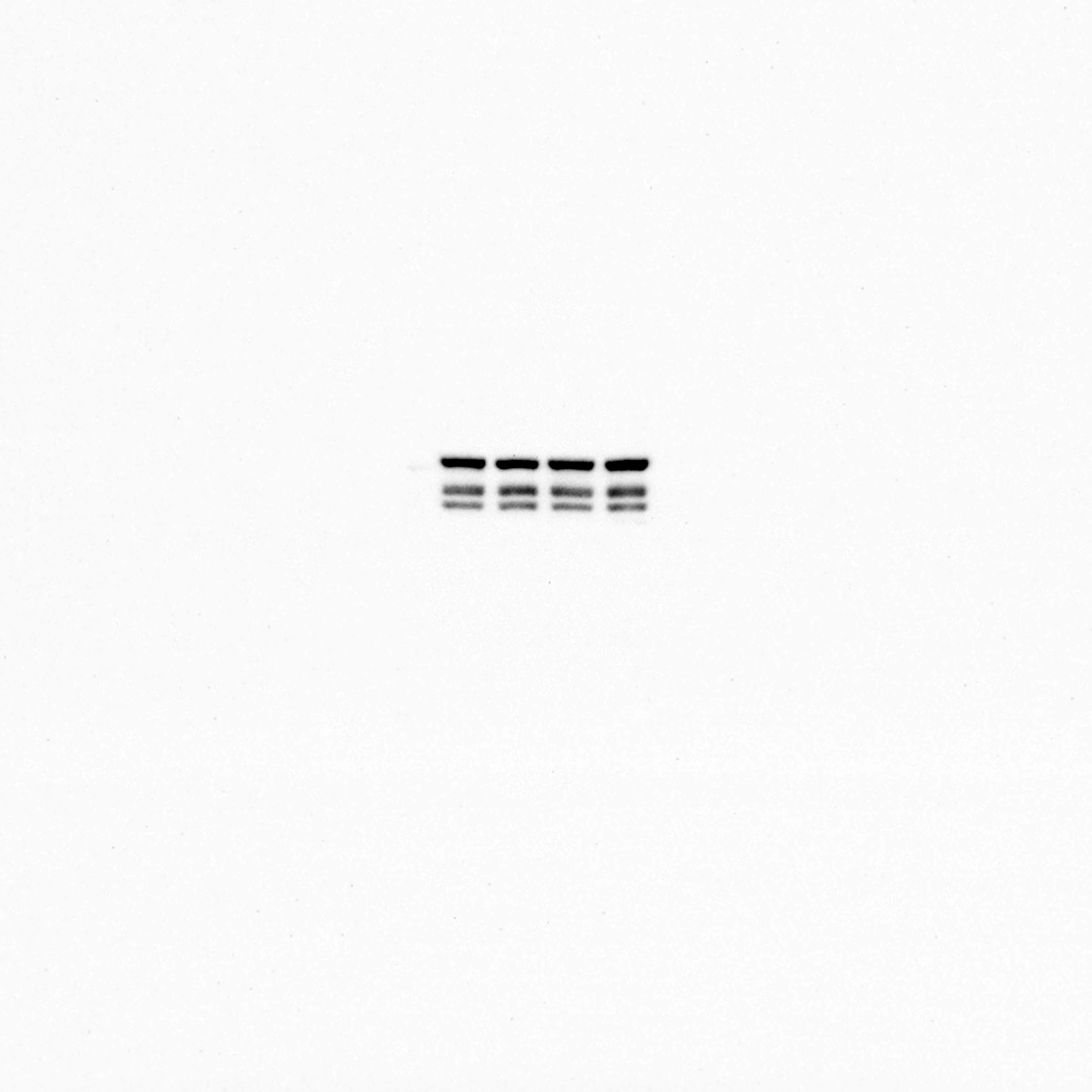


**90kD**

**Figure 5A**

Nuclear PKM2


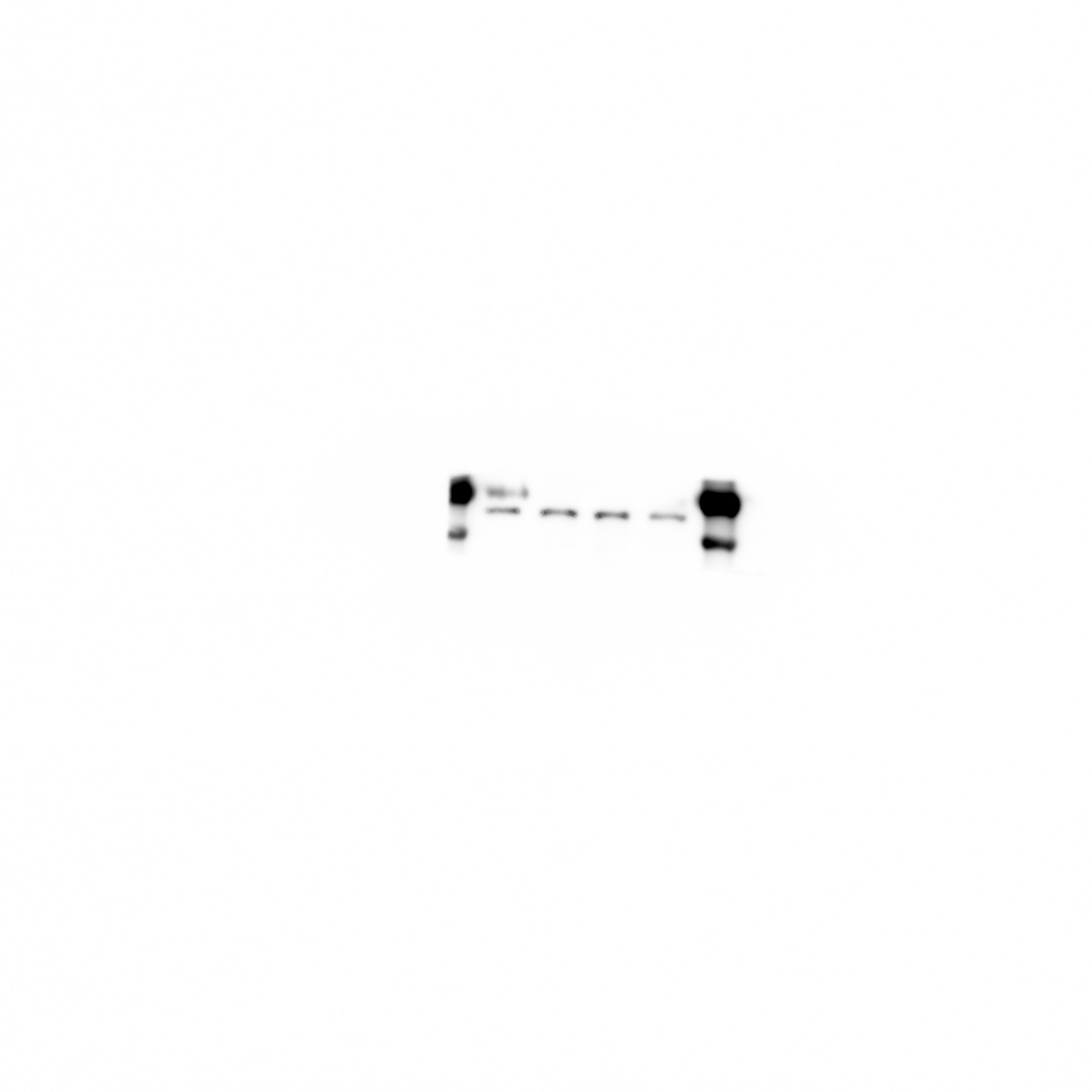


**60kD**

H3


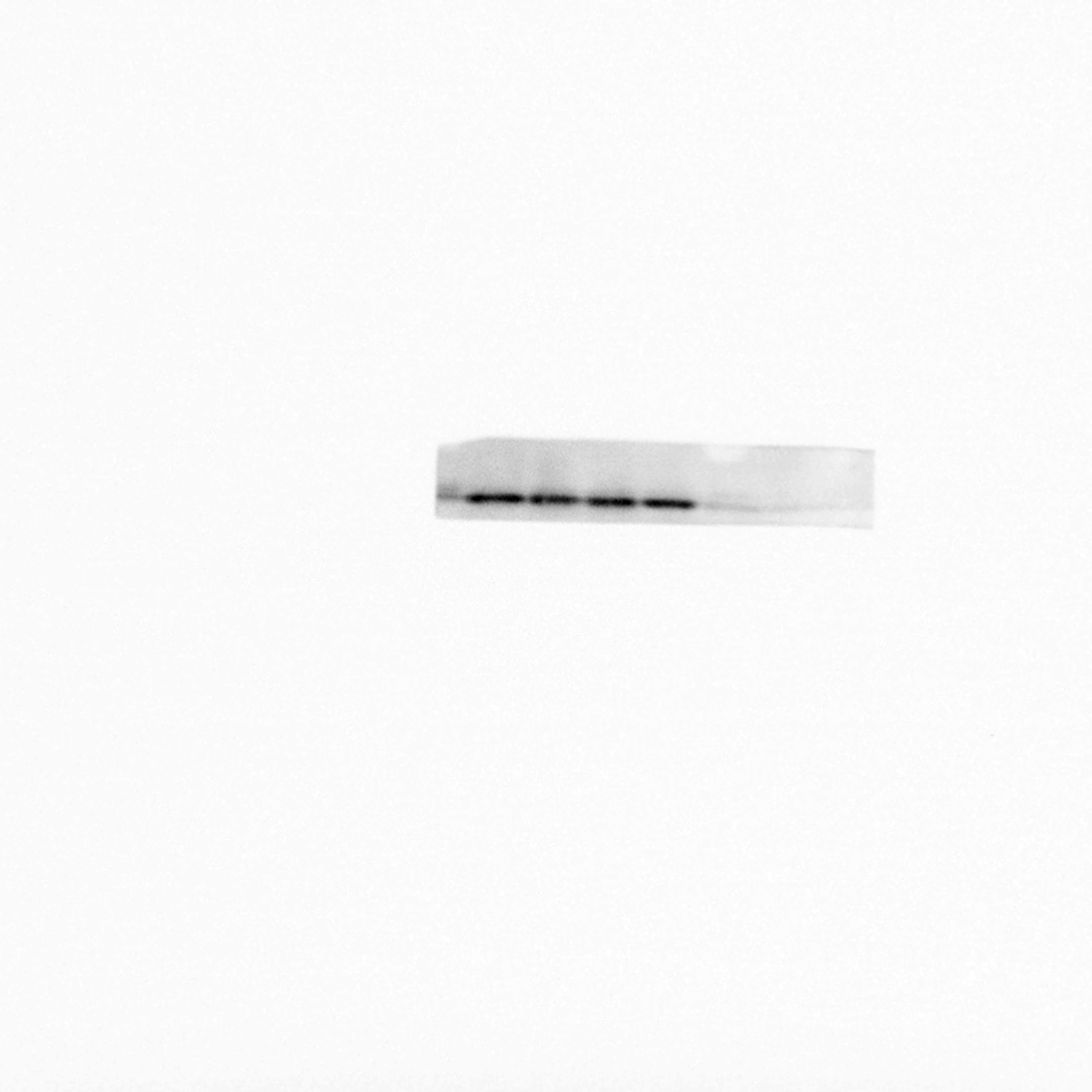


**17kD**

Cytoplasmic PKM2:


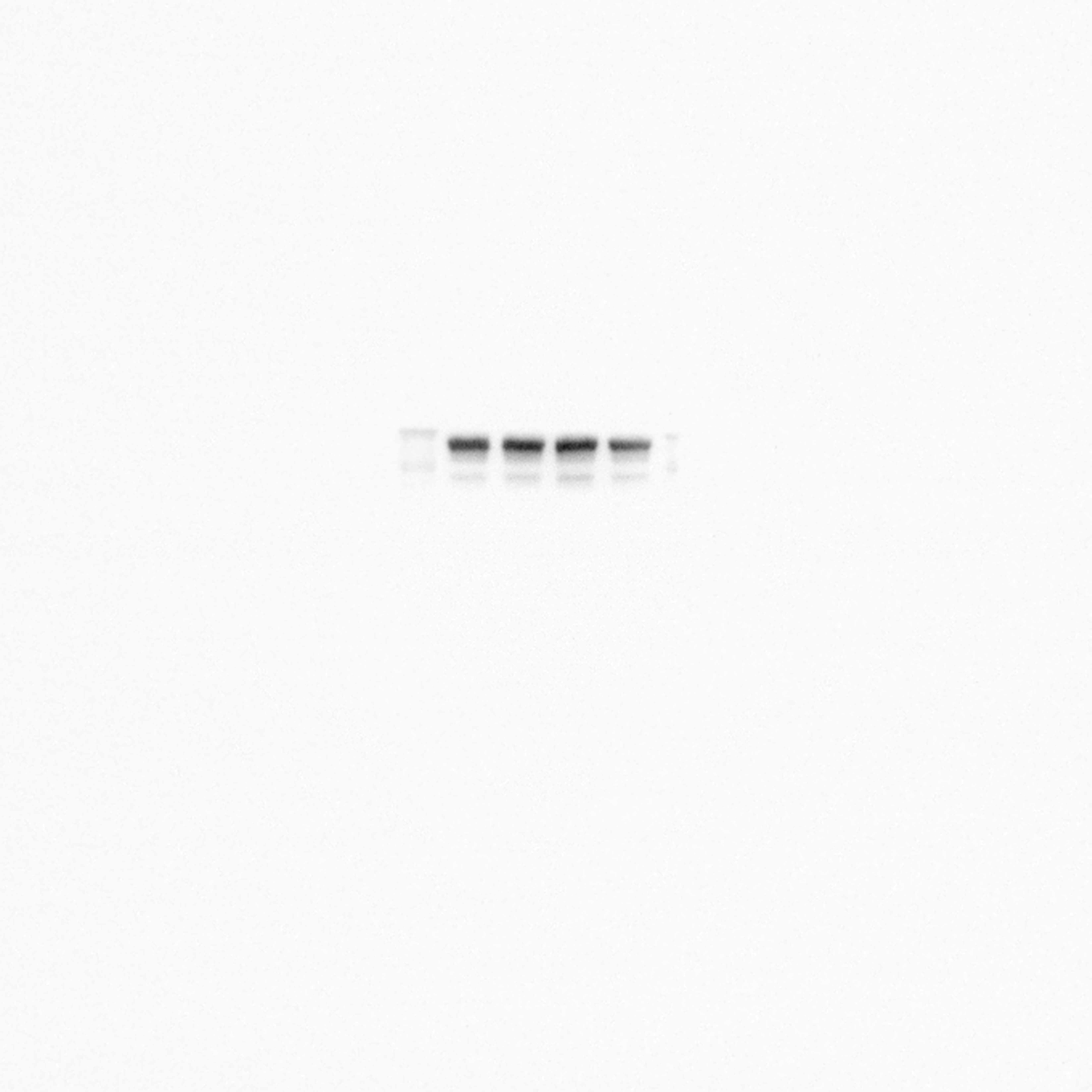


**60kD**

Hsp90


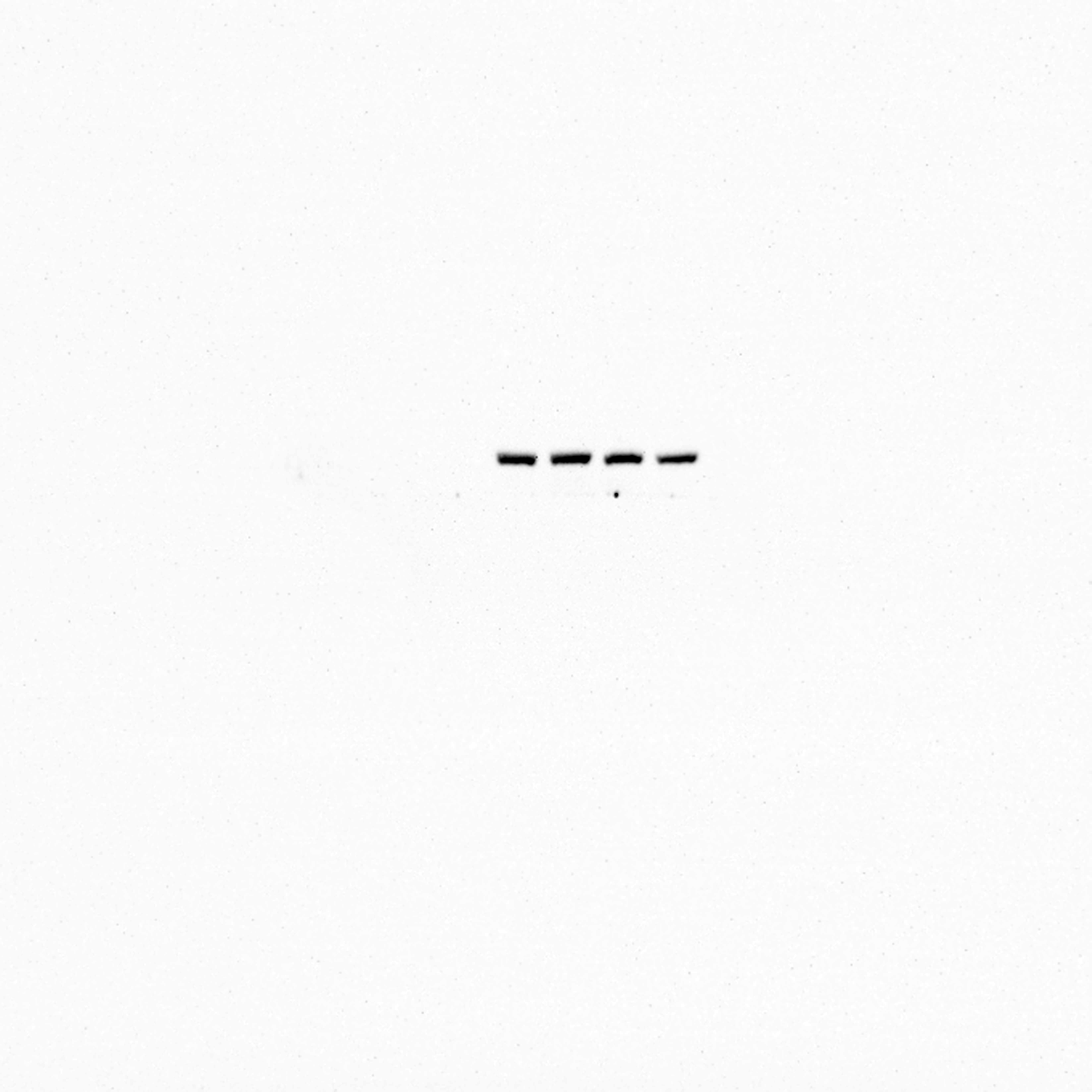


**90kD**

**Figure 5D**

STAT1


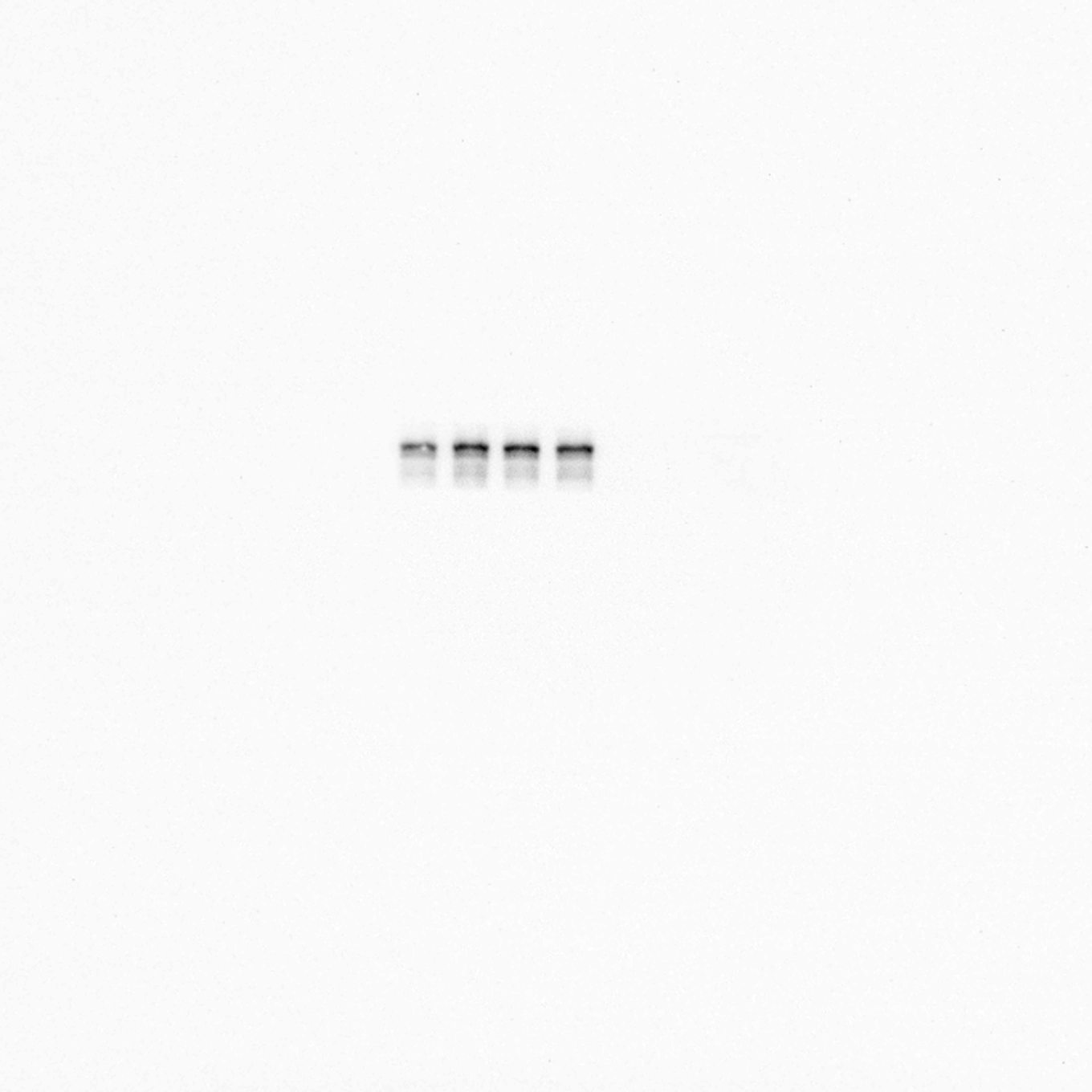


**91kD**

p-STAT1


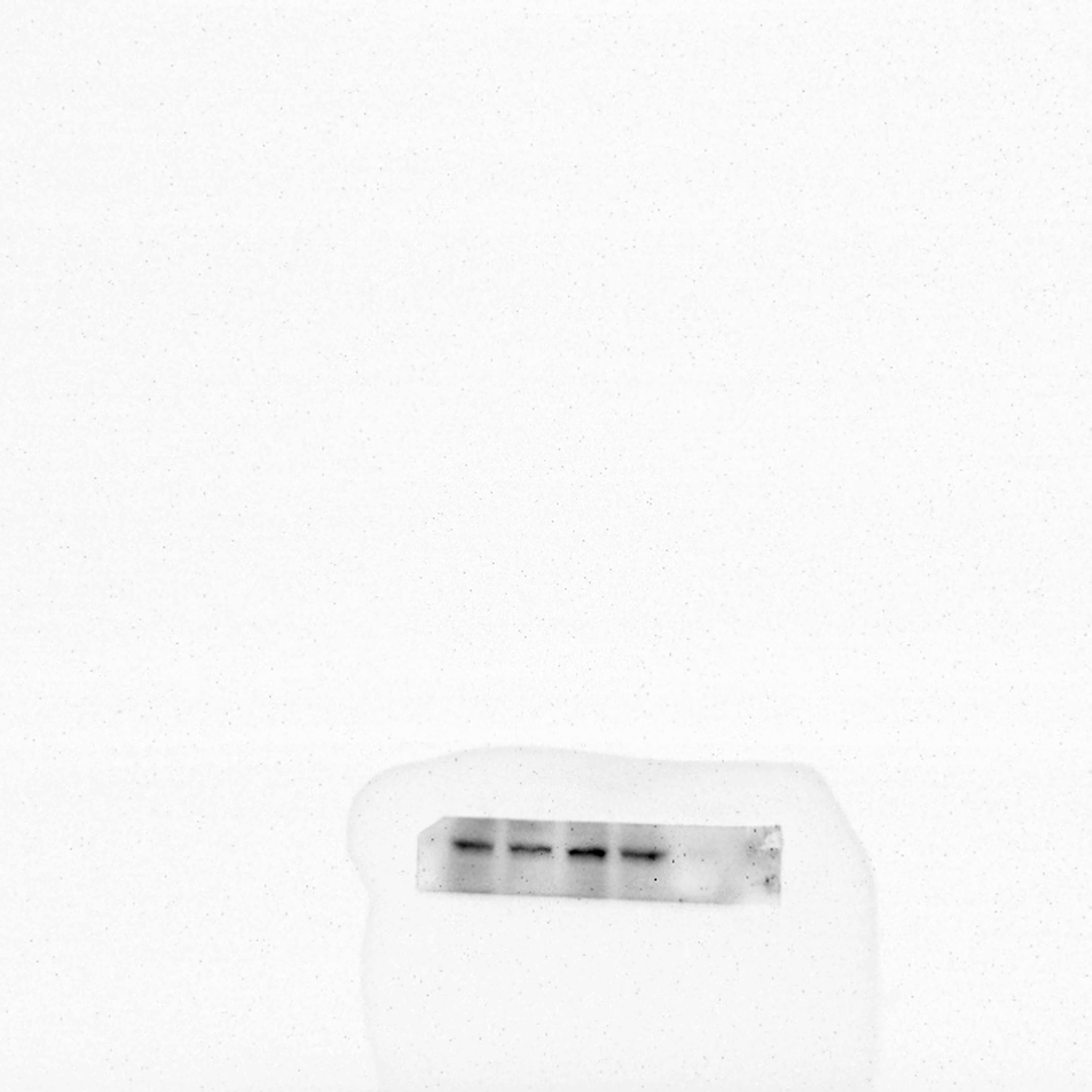


**91kD**

Actin


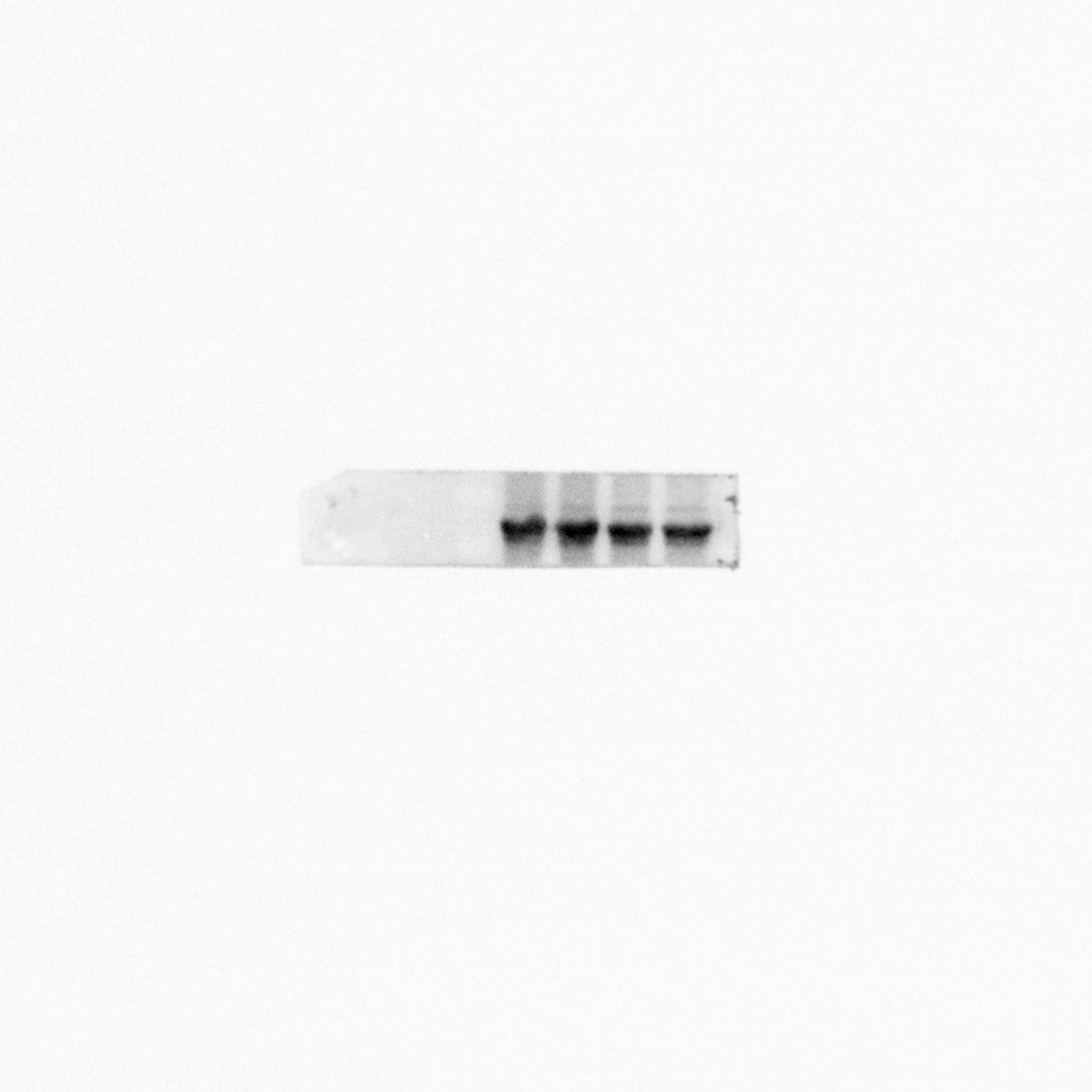


**42kD**

**Figure 5F**

STAT1


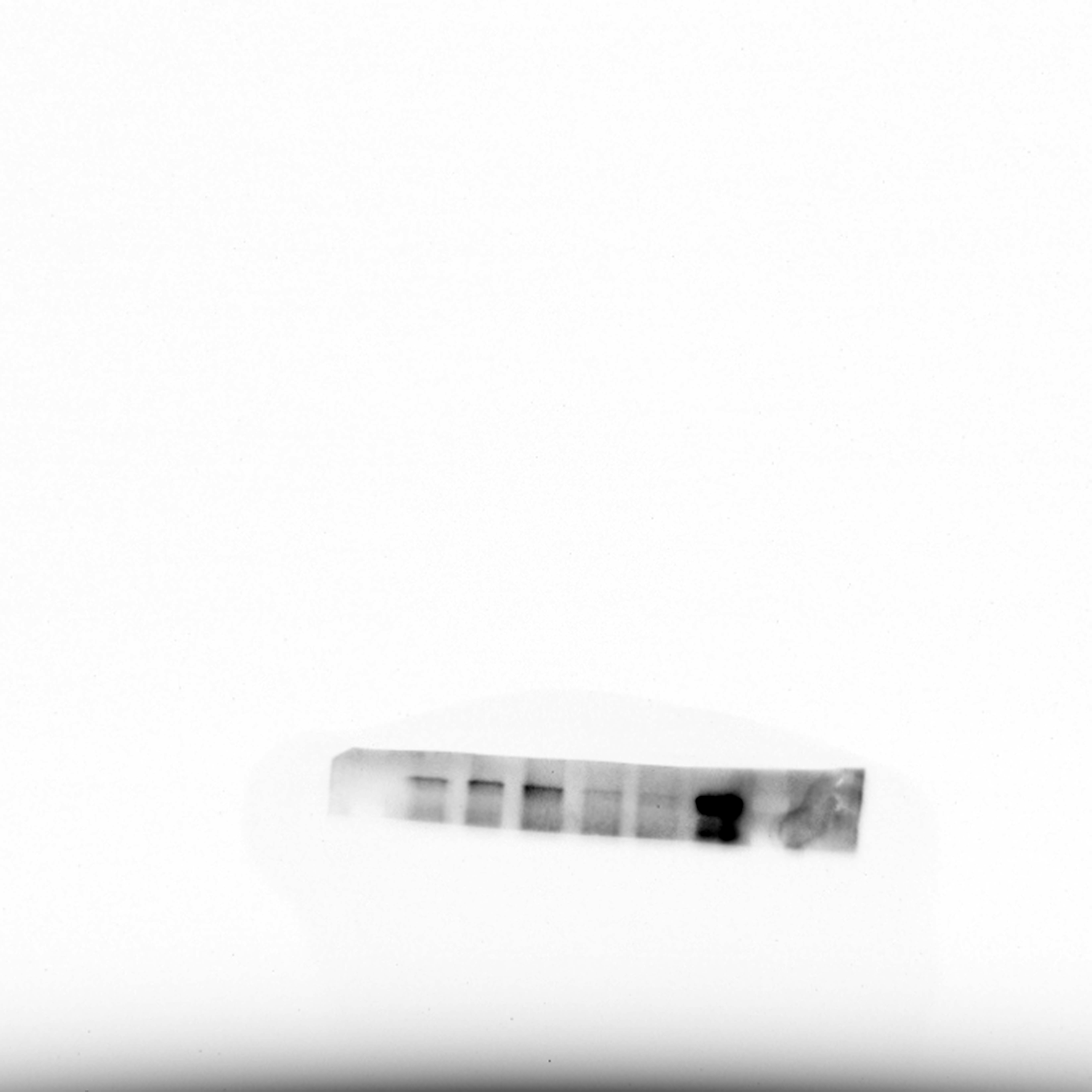


**91kD**

PKM2


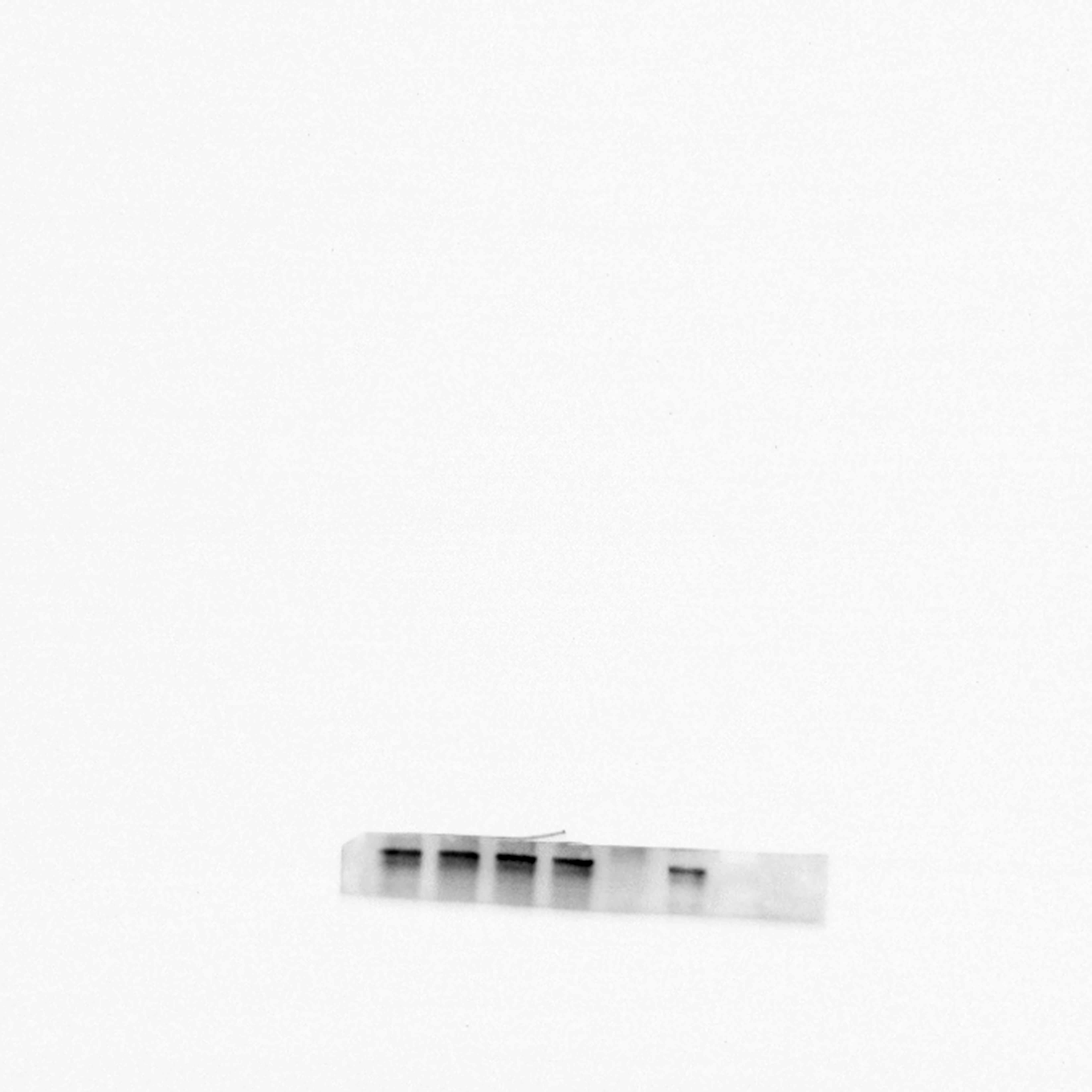


**60kD**

**Figure 6A**

STAT1


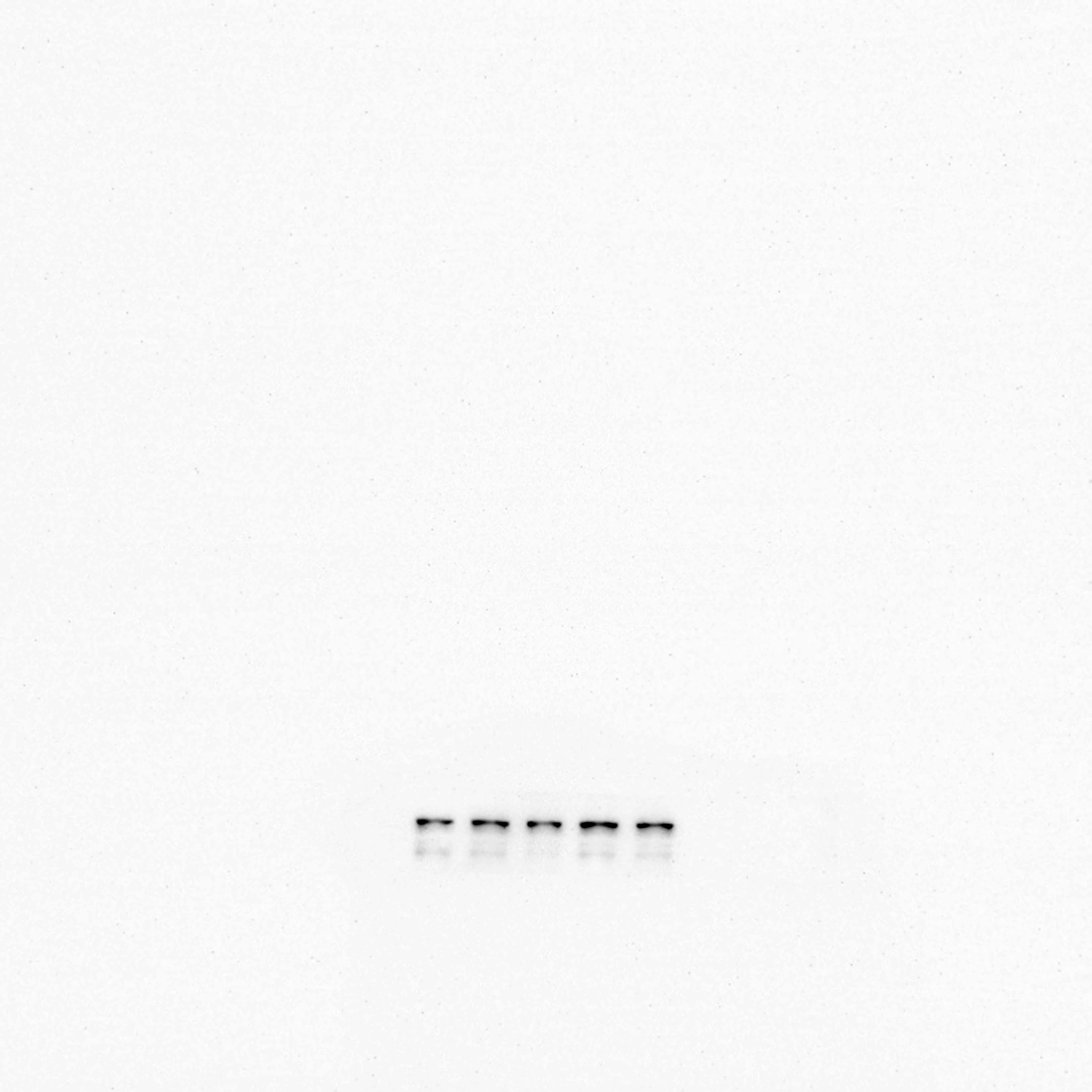


**91kD**

p-STAT1


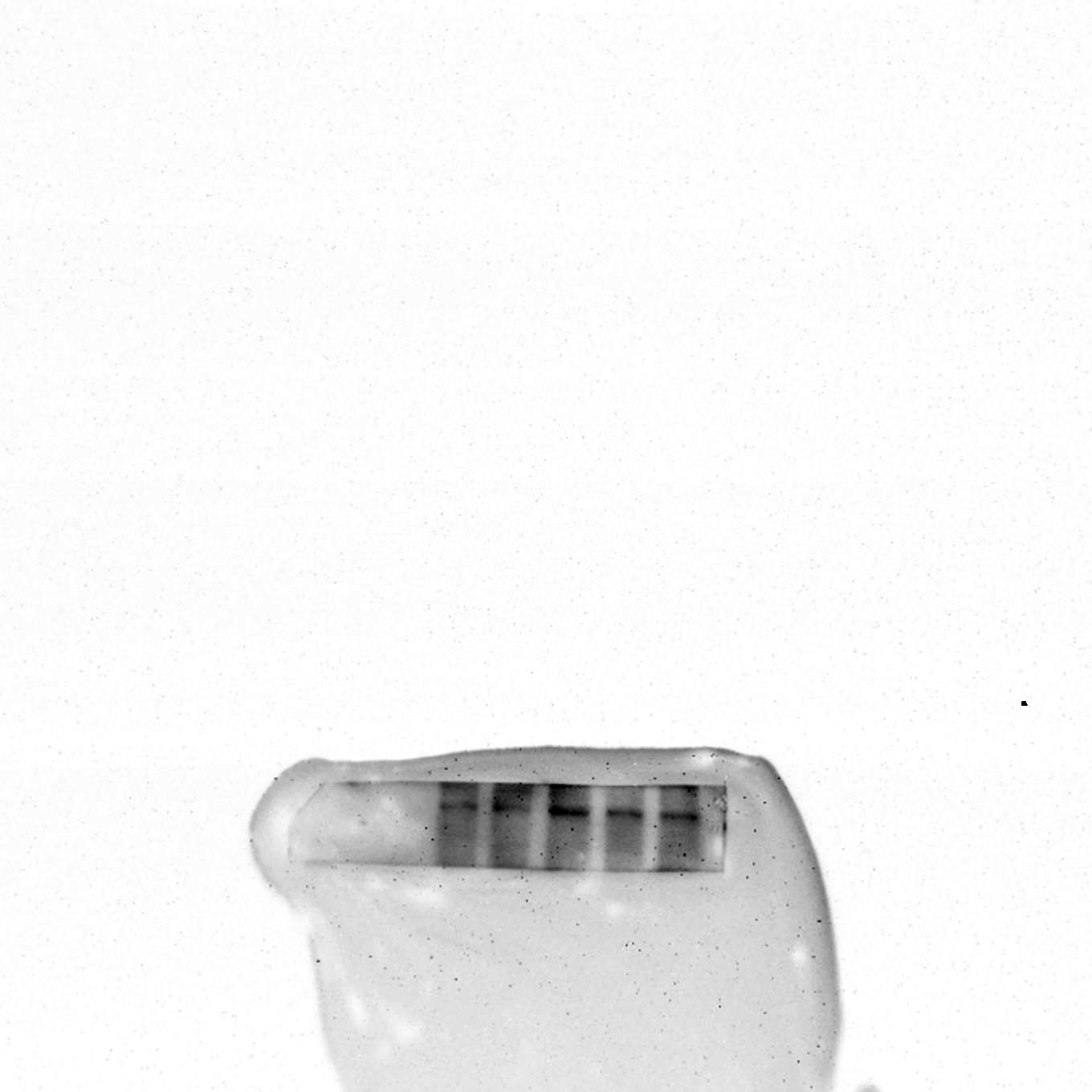


**91kD**

Actin


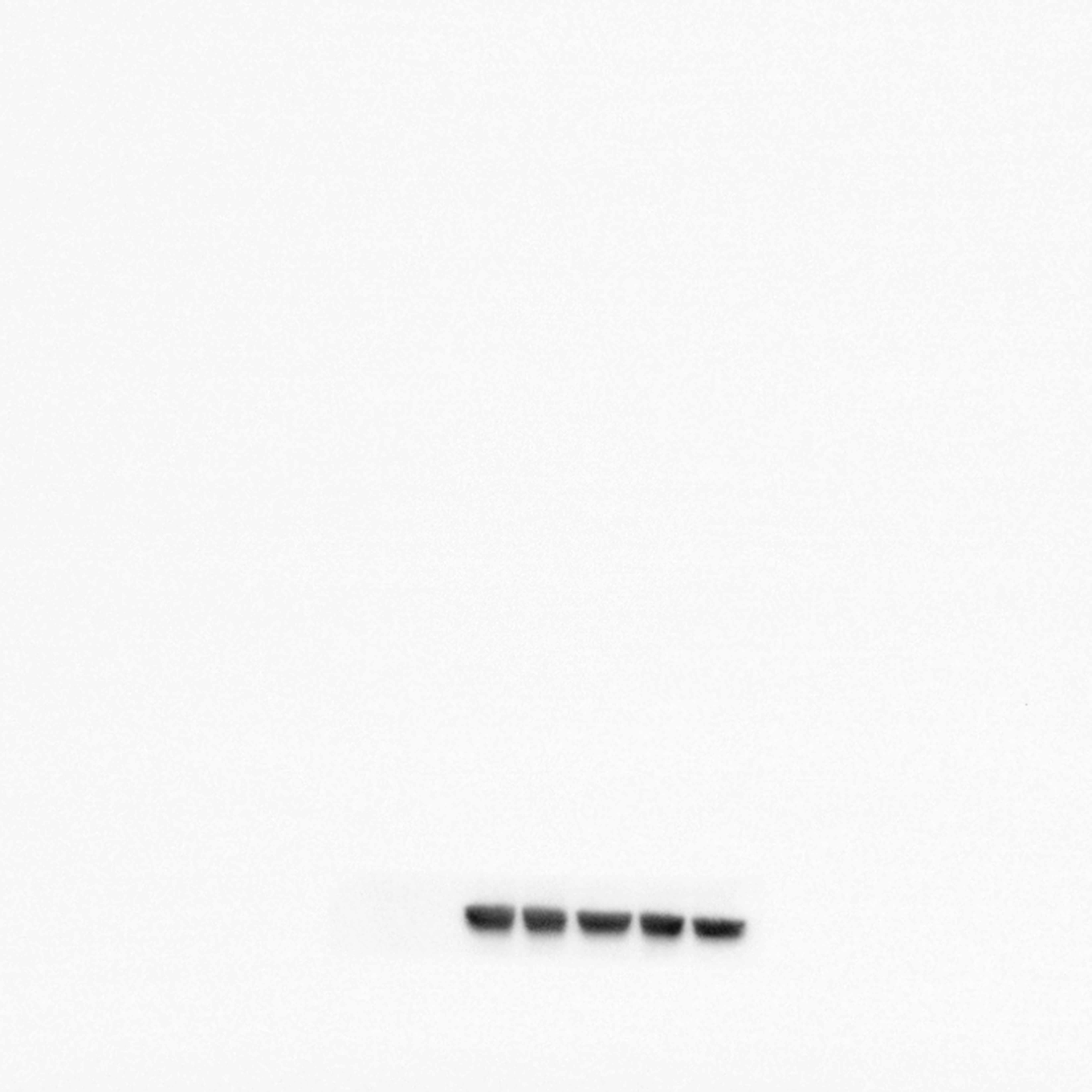


**60kD**

**Figure 6G**

Pro caspase3


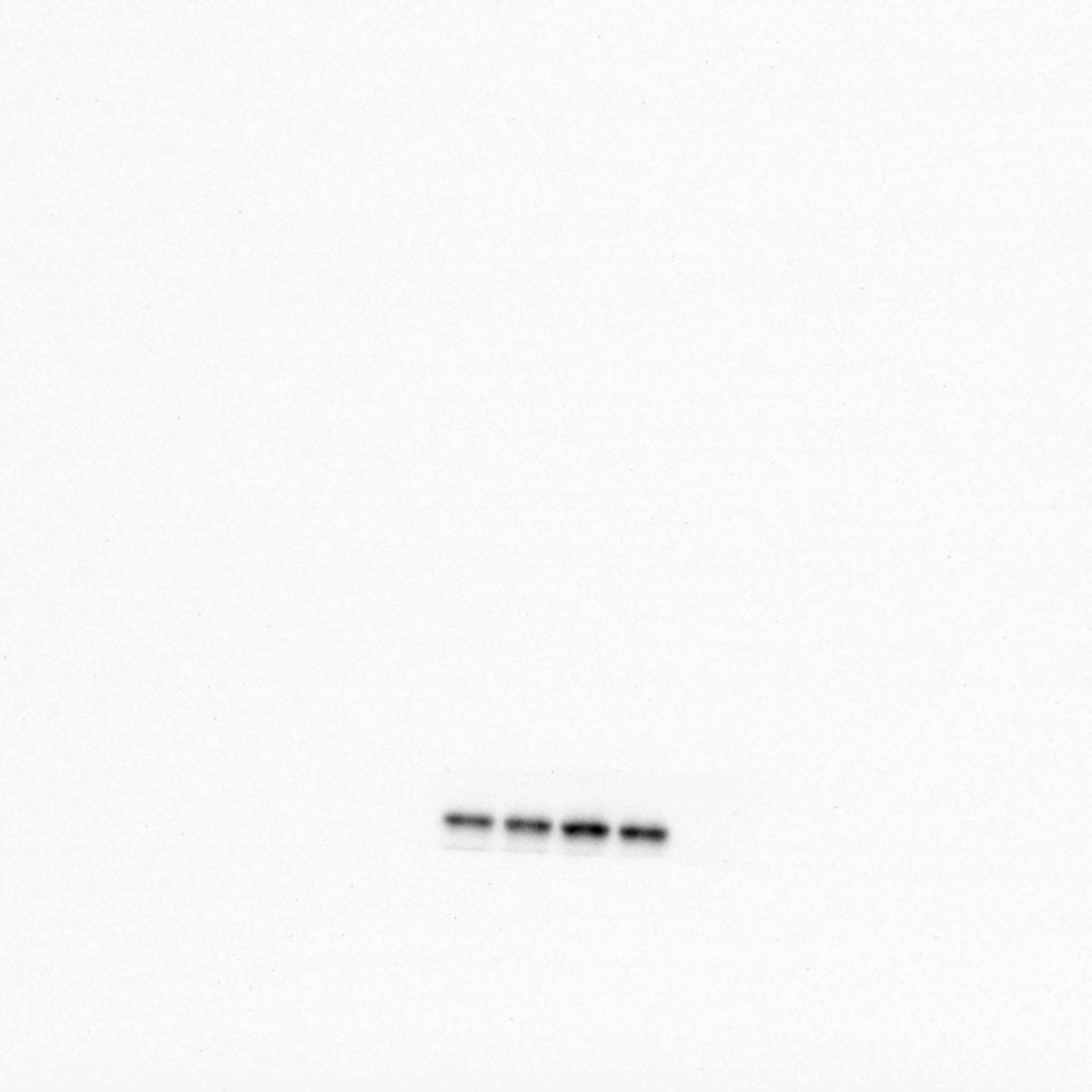


**35kD**

Cleaved caspase3


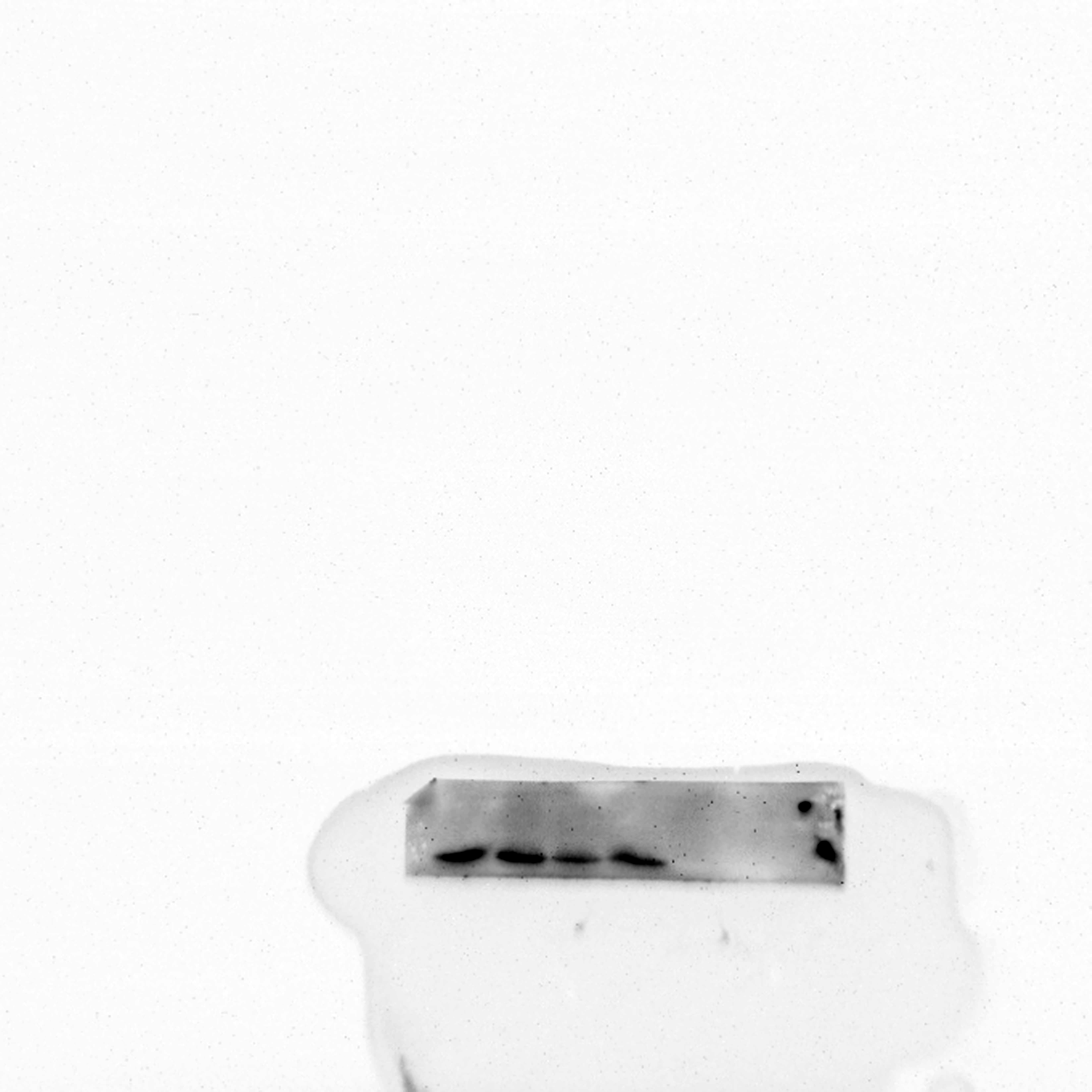


**15kD**

Mcl-1


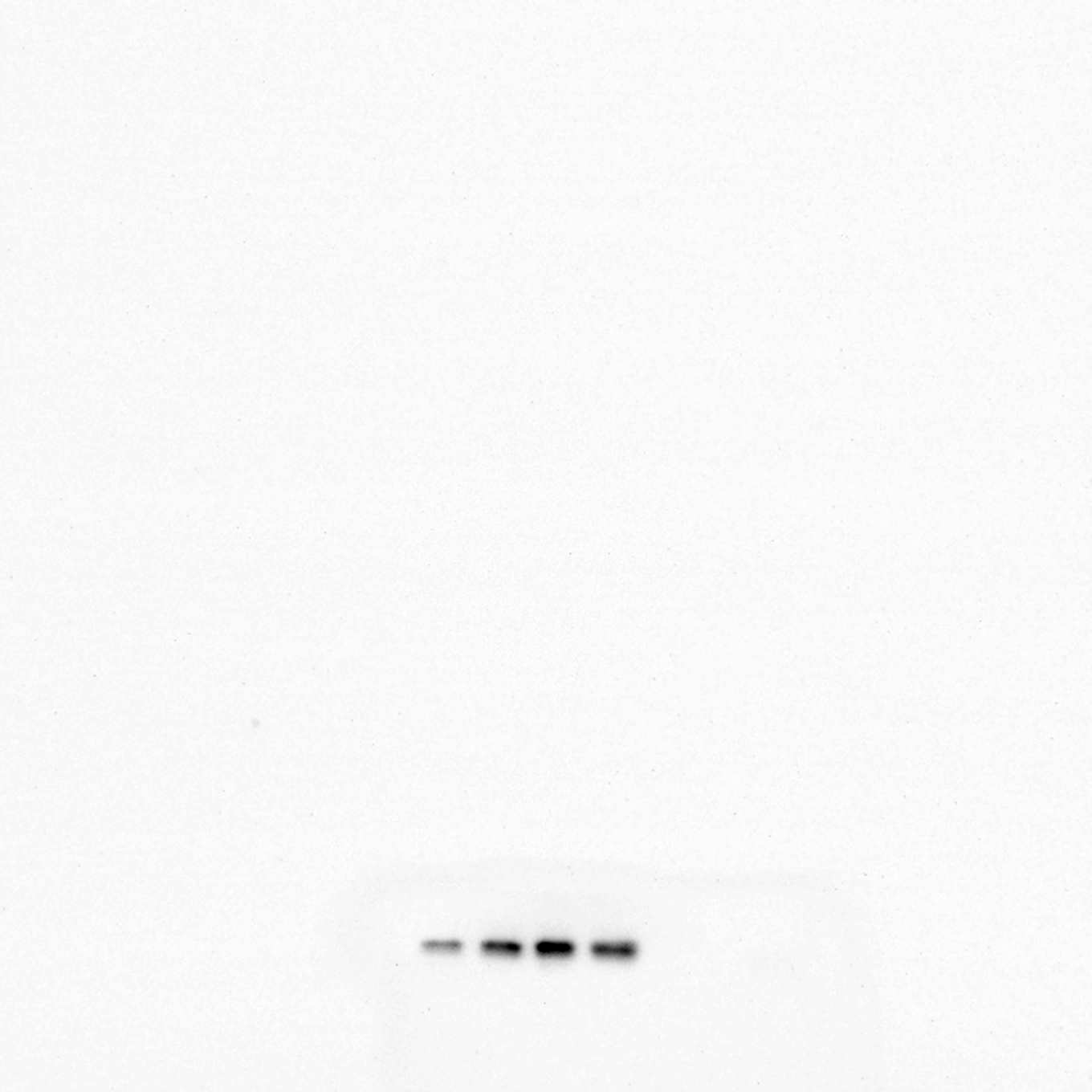


**40kD**

Hsp90


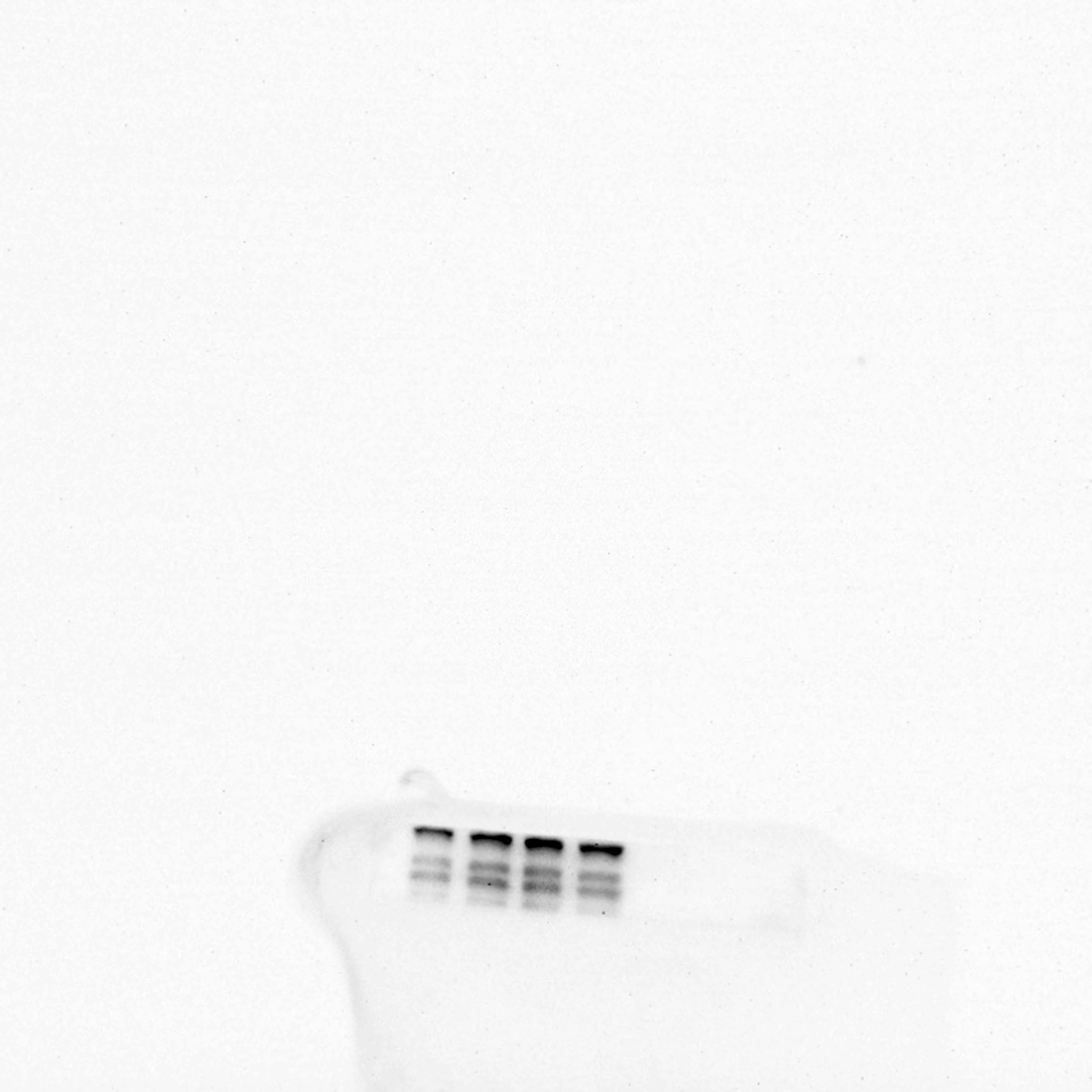


**90kD**
